# Supplementary material for: Claudin-low-like mouse mammary tumors show distinct transcriptomic patterns uncoupled from genomic drivers
Source: Breast Cancer Res. 2019 Jul 31;21:85. doi: 10.1186/s13058-019-1170-8 (PMC6670237; doi:10.1186/s13058-019-1170-8)
Supplement: Supplementary file 7 — The mutational spectra and mutational signatures of MPA/DMBA-induced mammary tumors. a T>A transversions were the most frequent mutation type in MPA/DMBA-induced tumors, followed by C>A transversions. b Heatmap of mutational frequencies by trinucleotide context. There was an overrepresentation of T>N mutations in positions with a 3′ guanine and C>N mutations in positions with a 3′ adenine. c Histogram of C>A and T>A transversions by trinucleotide context in a representative tumor (S159_14_8). d Mutation signature 22 was the predominant mutational signature in the MPA/DMBA-induced tumors and was evident in all tumors in the cohort. (PDF 214 kb) [file 13058_2019_1170_MOESM7_ESM.pdf]

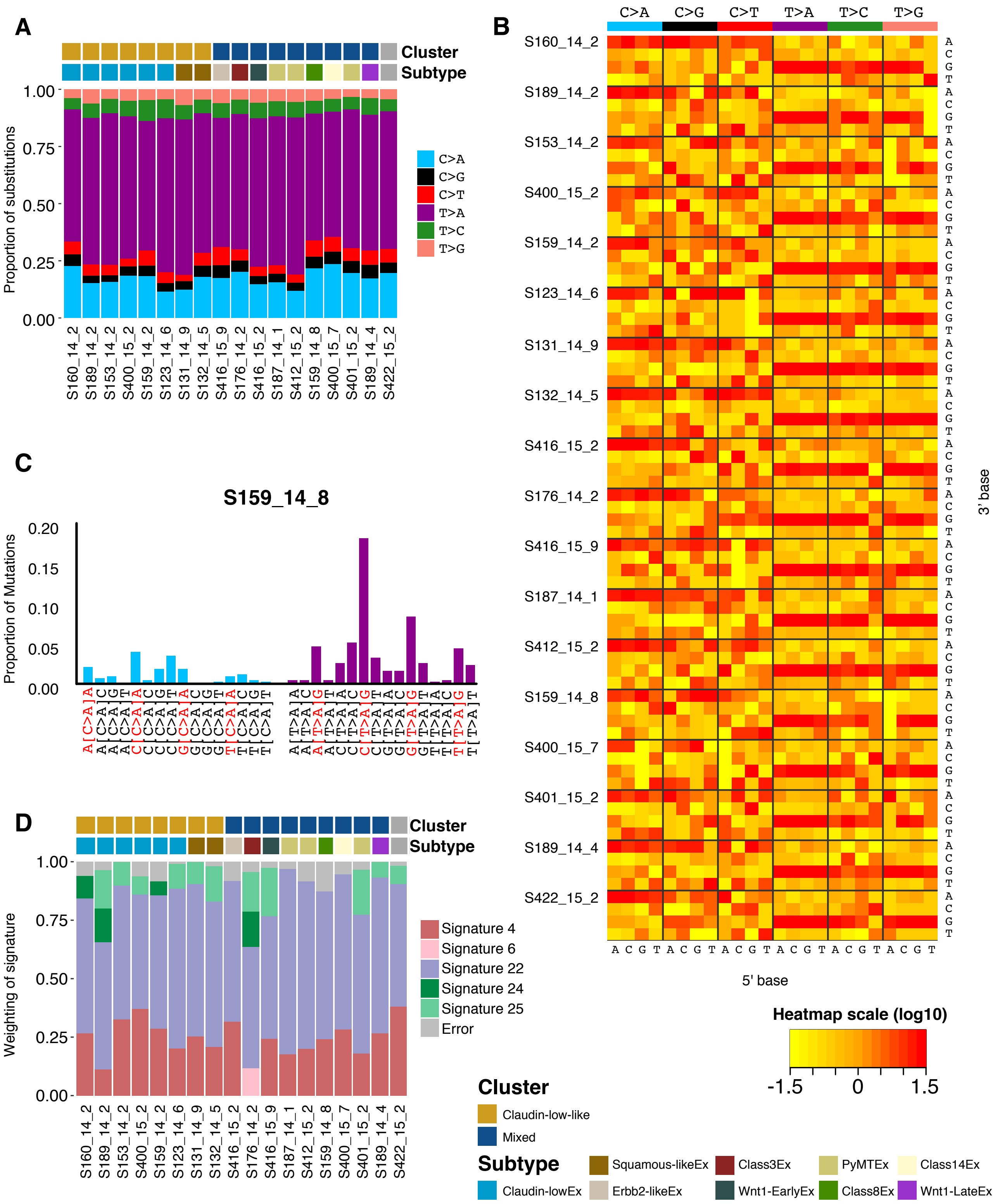
A
Cluster
Subtype
1.00
0.75
0.50
0.25
0.00
Proportion of substitutions
C>A
C>G
C>T
T>A
T>C
T>G
S160_14_2
S189_14_2
S153_14_2
S400_15_2
S159_14_2
S123_14_6
S131_14_9
S132_14_5
S416_15_9
S176_14_2
S416_15_2
S187_14_1
S412_15_2
S159_14_8
S400_15_7
S401_15_2
S189_14_4
S422_15_2
B
C>A
C>G
C>T
T>A
T>C
T>G
S160_14_2
S189_14_2
S153_14_2
S400_15_2
S159_14_2
S123_14_6
S131_14_9
S132_14_5
S416_15_2
S176_14_2
S416_15_9
S187_14_1
S412_15_2
S159_14_8
S400_15_7
S401_15_2
S189_14_4
S422_15_2
3' base
5' base
A C G T A C G T A C G T A C G T A C G T A C G T
Heatmap scale (log10)
-1.5
0
1.5
C
S159_14_8
Proportion of Mutations
0.20
0.15
0.10
0.05
0.00
D
Cluster
Subtype
Weighting of signature
Signature 4
Signature 6
Signature 22
Signature 24
Signature 25
Error
Cluster
Claudin-low-like
Mixed
Subtype
Claudin-lowEx
Squamous-likeEx
Erbb2-likeEx
Class3Ex
Wnt1-EarlyEx
PyMTEx
Class8Ex
Class14Ex
Wnt1-LateEx
