# Supplementary figures and images for: Claudin-low-like mouse mammary tumors show distinct transcriptomic patterns uncoupled from genomic drivers
Source: Breast Cancer Res. 2019 Jul 31;21:85. doi: 10.1186/s13058-019-1170-8 (PMC6670237; doi:10.1186/s13058-019-1170-8)

S123\_14\_6

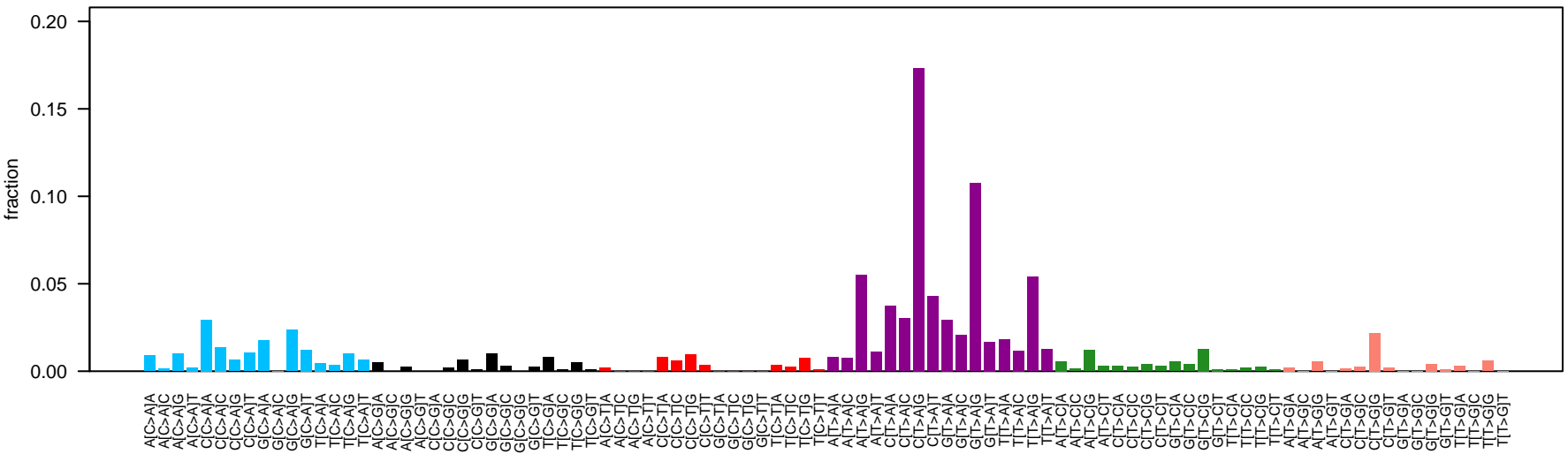

Signature.4 : 0.202 & Signature.22 : 0.683 & Signature.25 : 0.106

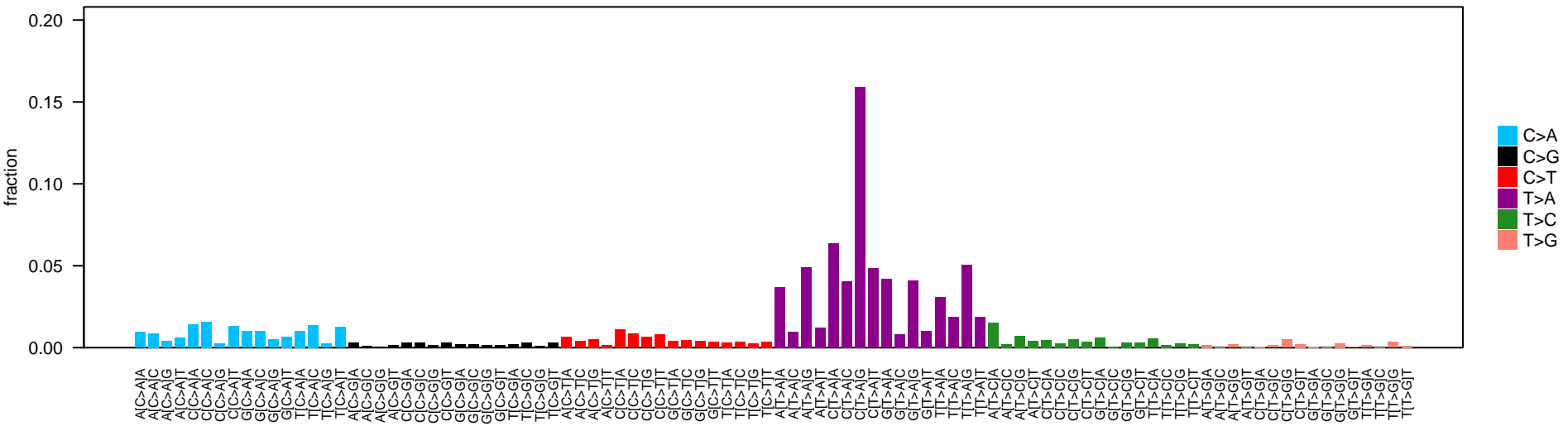

error = 0.095

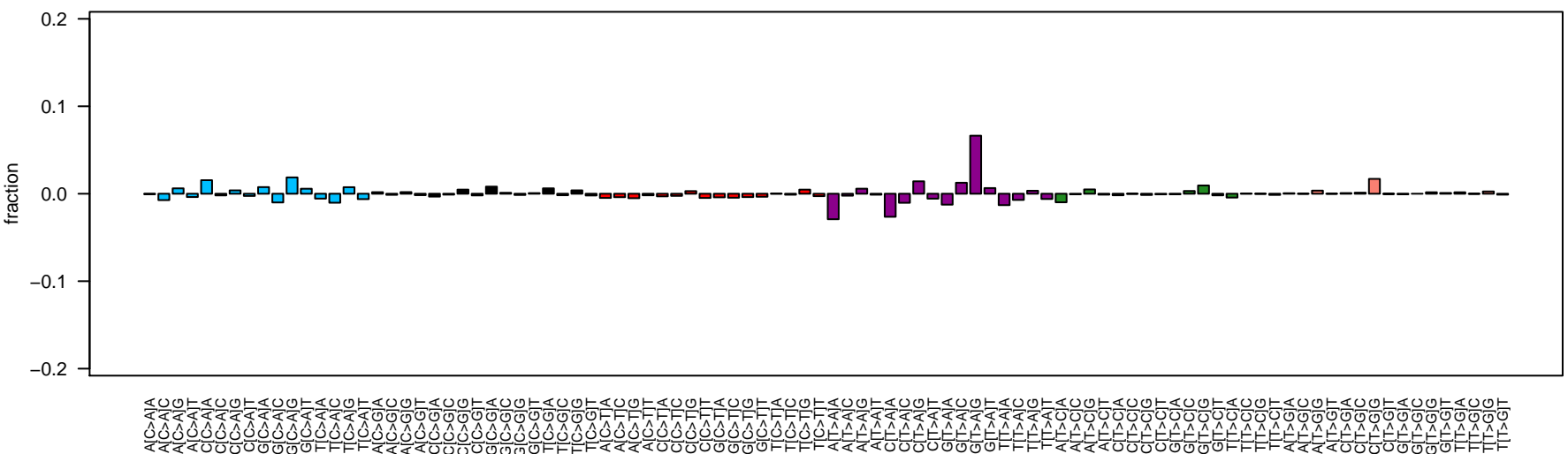

Supplement: Supplementary file 8 — Mutational signatures for all MPA/DMBA-induced tumors. (ZIP 142 kb) [file 13058_2019_1170_MOESM8_ESM.zip › S123_14_6.pdf]

S131\_14\_9

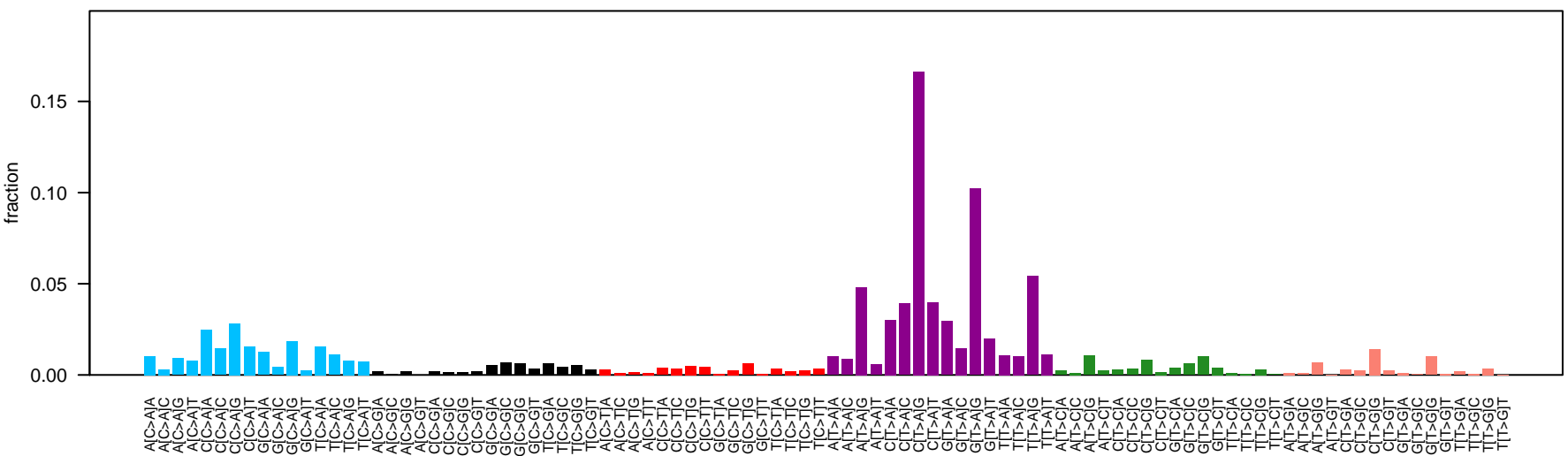

Signature.4 : 0.254 & Signature.22 : 0.652 & Signature.25 : 0.094

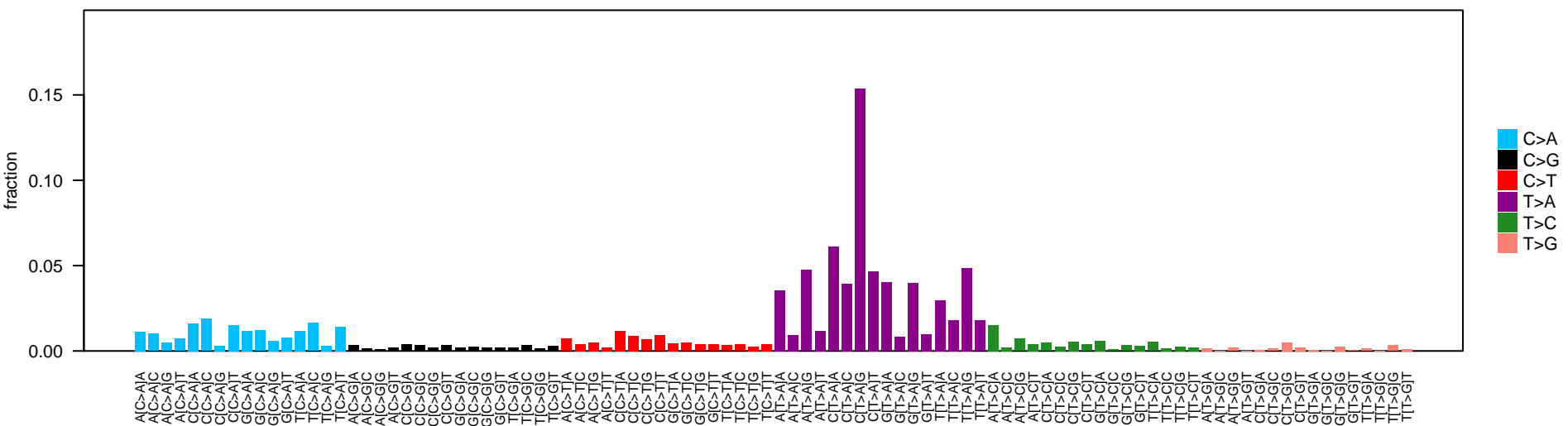

error = 0.092

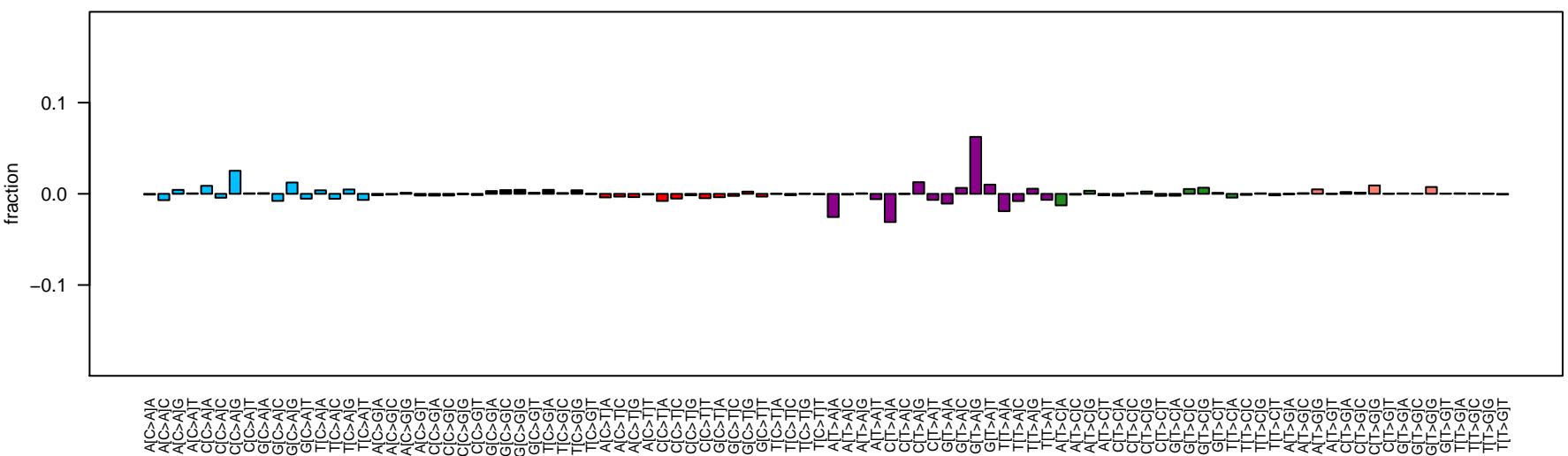

Supplement: Supplementary file 8 — Mutational signatures for all MPA/DMBA-induced tumors. (ZIP 142 kb) [file 13058_2019_1170_MOESM8_ESM.zip › S131_14_9.pdf]

S132\_14\_5

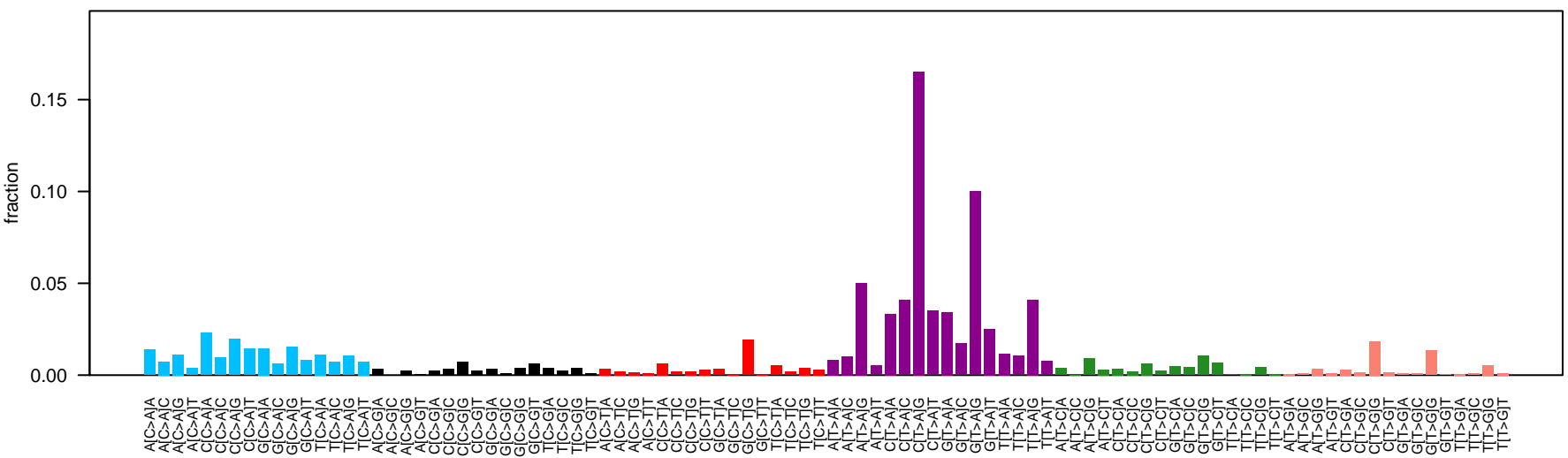

Signature.4 : 0.209 & Signature.22 : 0.622 & Signature.25 : 0.151

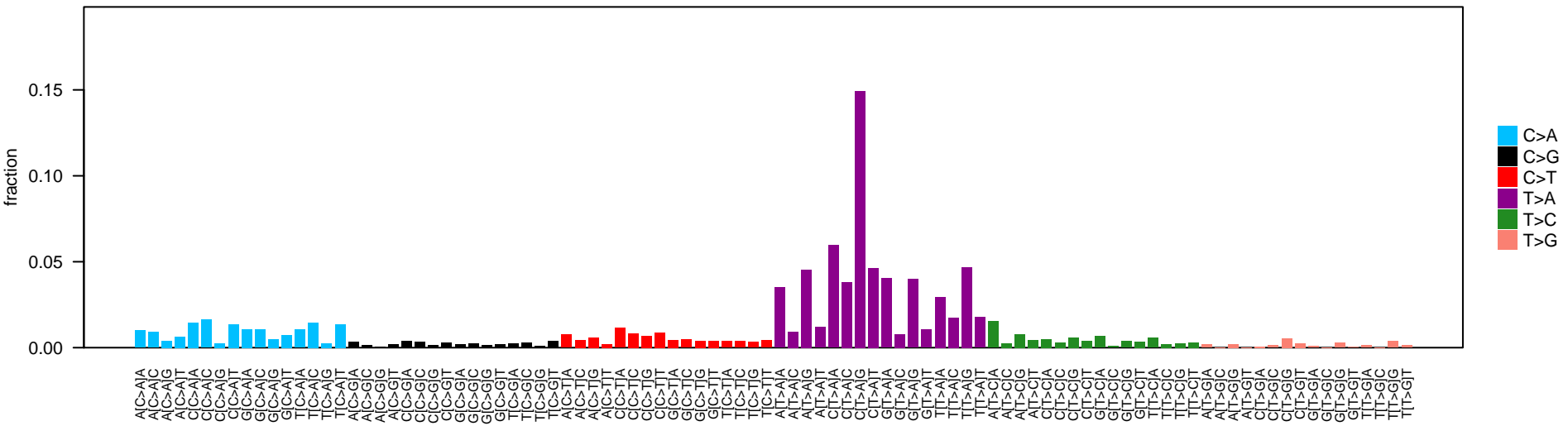

error = 0.09

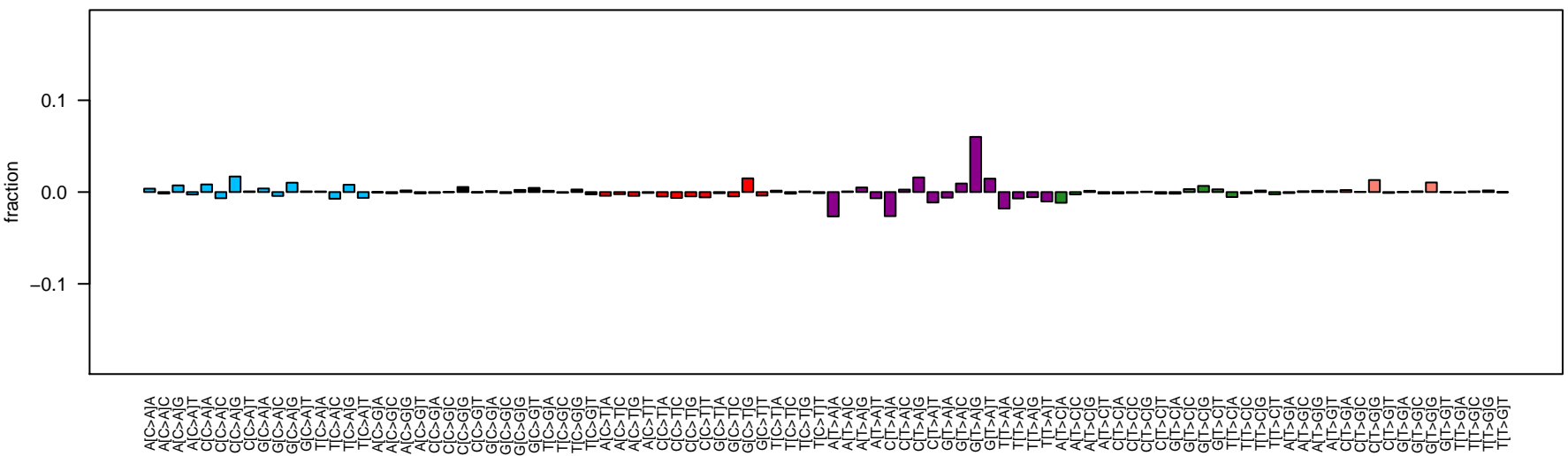

Supplement: Supplementary file 8 — Mutational signatures for all MPA/DMBA-induced tumors. (ZIP 142 kb) [file 13058_2019_1170_MOESM8_ESM.zip › S132_14_5.pdf]

S159\_14\_2

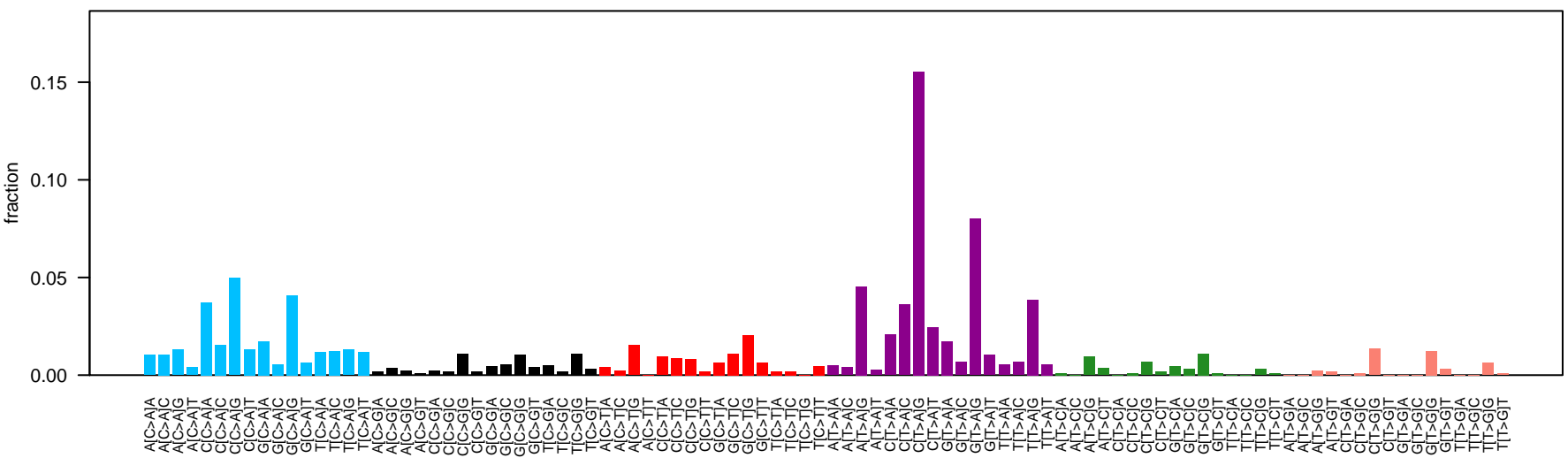

Signature.4 : 0.287 & Signature.22 : 0.57 & Signature.24 : 0.06

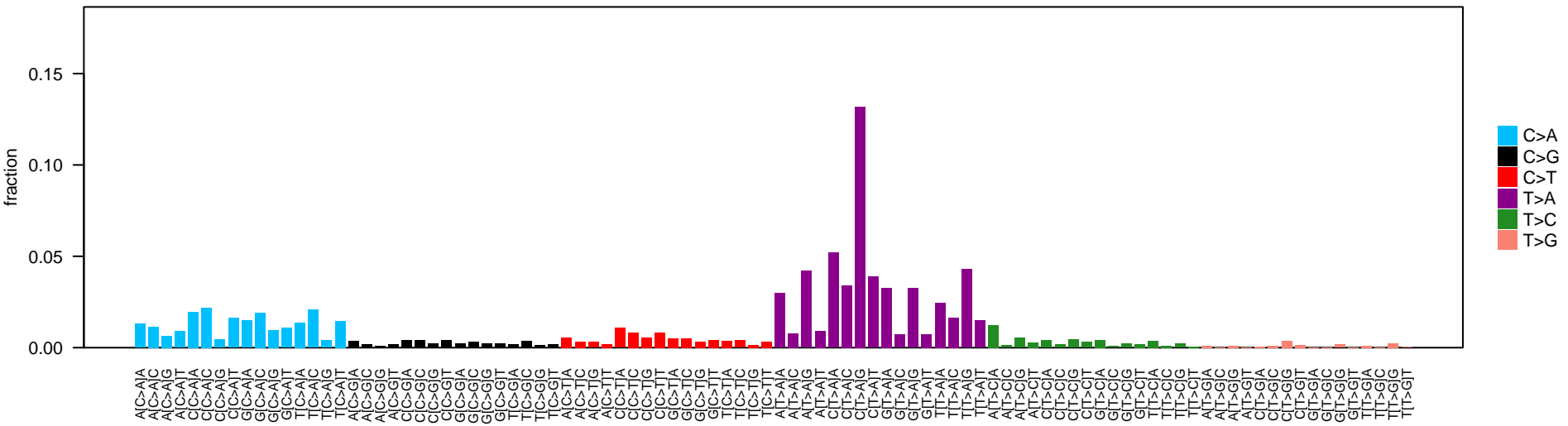

error = 0.103

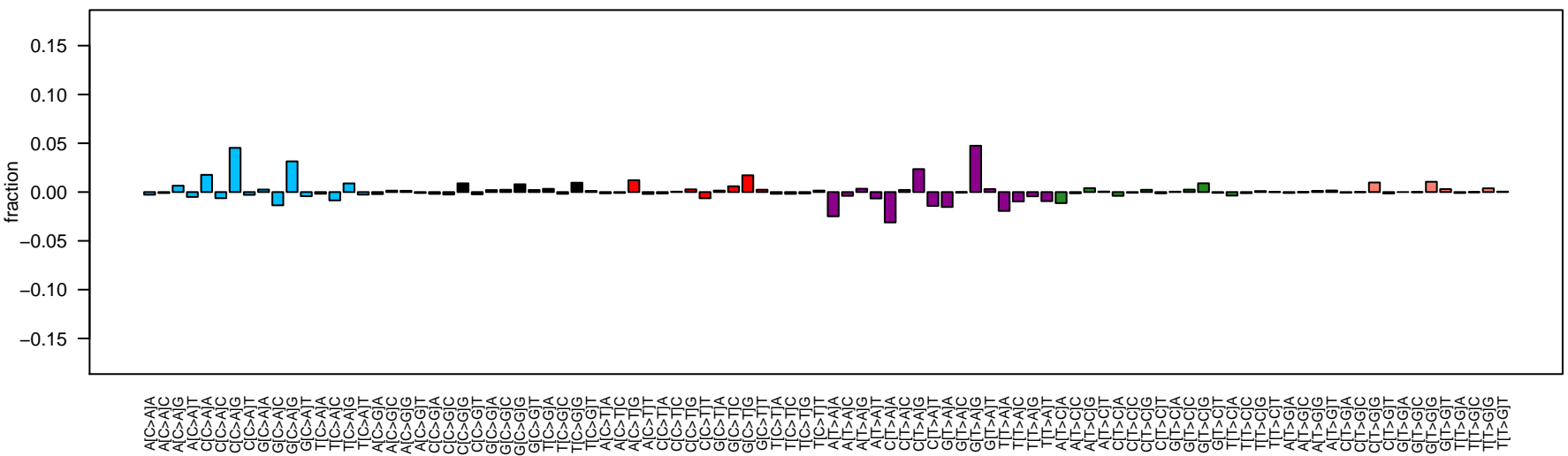

Supplement: Supplementary file 8 — Mutational signatures for all MPA/DMBA-induced tumors. (ZIP 142 kb) [file 13058_2019_1170_MOESM8_ESM.zip › S159_14_2.pdf]

S159\_14\_8

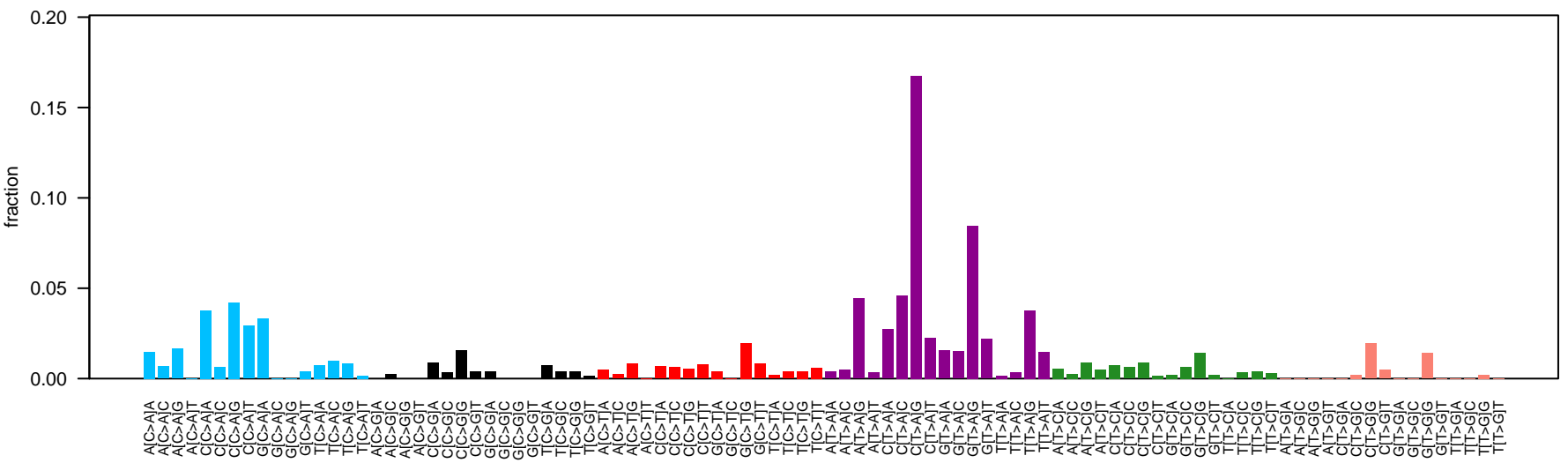

Signature.4 : 0.242 & Signature.22 : 0.631

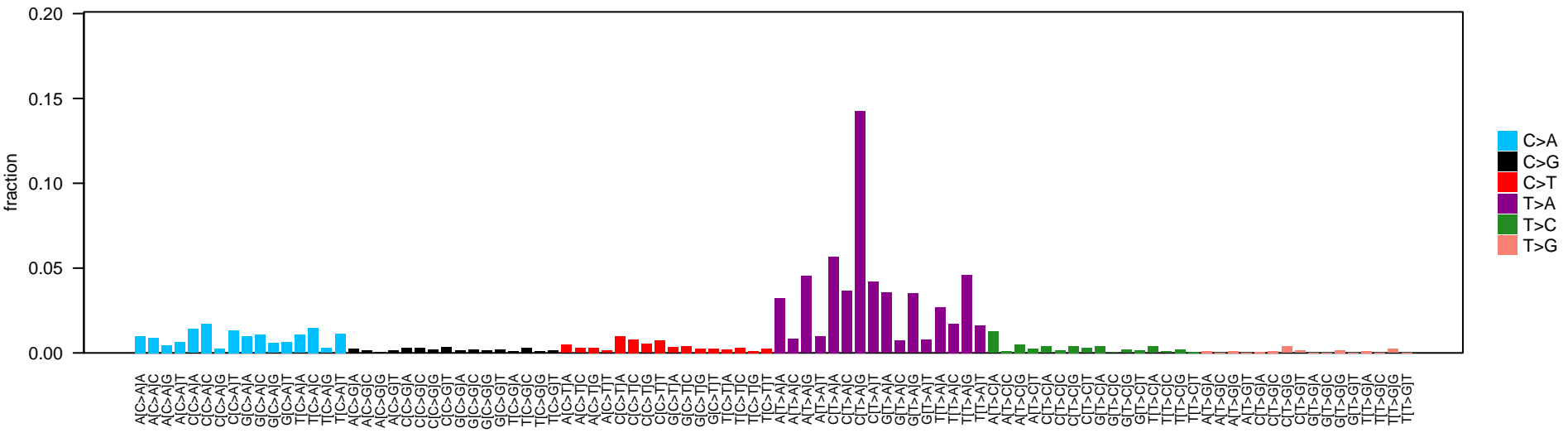

error = 0.109

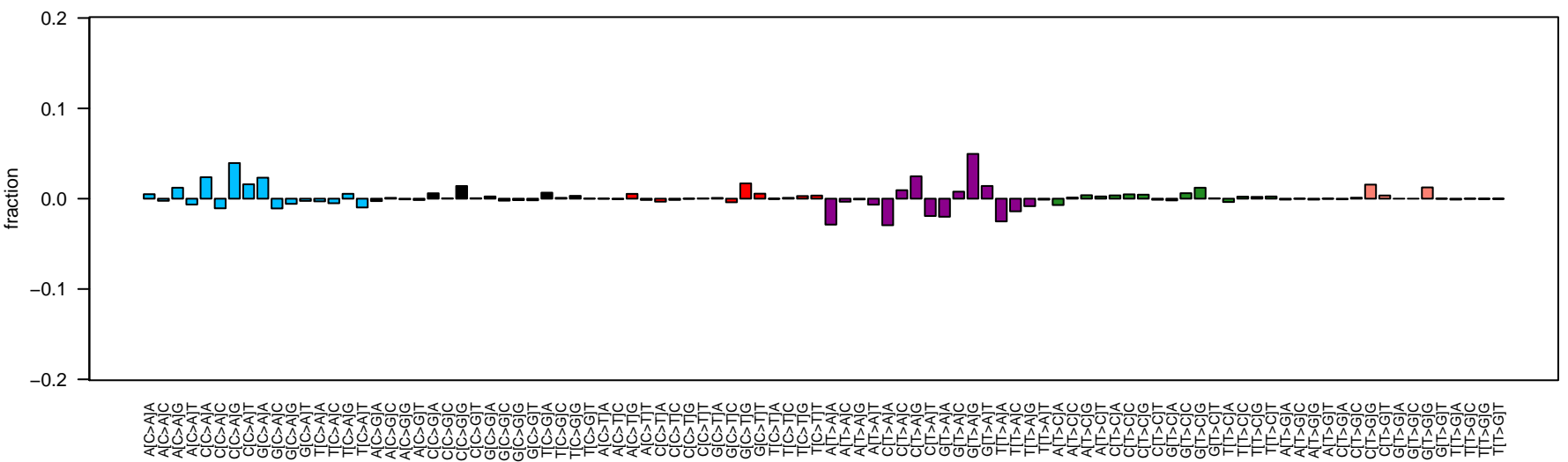

Supplement: Supplementary file 8 — Mutational signatures for all MPA/DMBA-induced tumors. (ZIP 142 kb) [file 13058_2019_1170_MOESM8_ESM.zip › S159_14_8.pdf]

S160\_14\_2

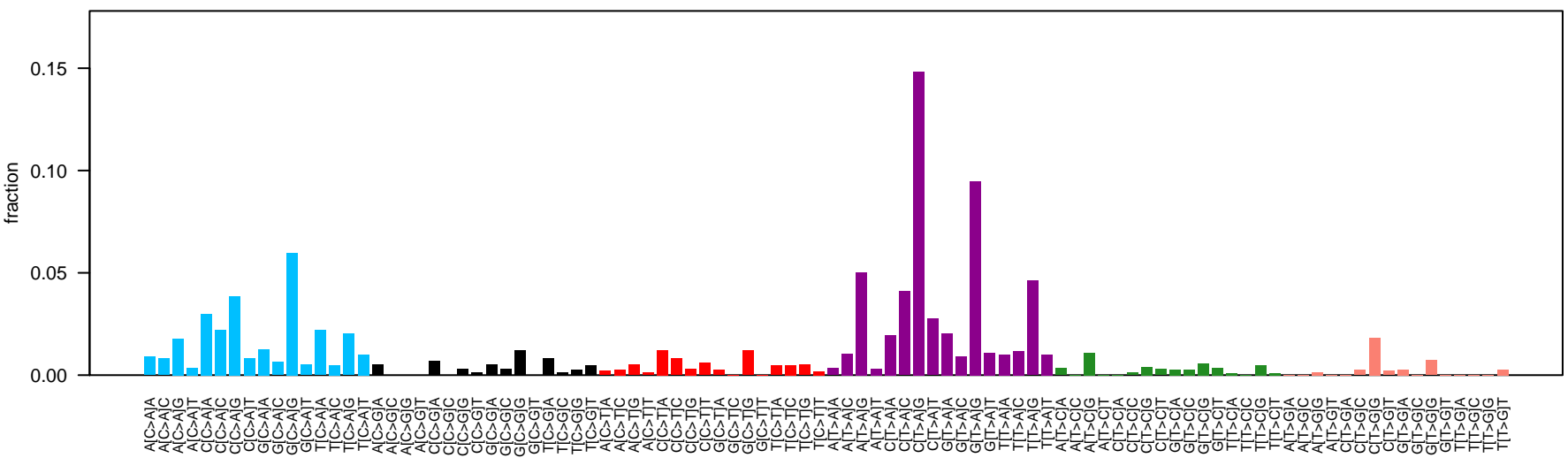

Signature.4 : 0.267 & Signature.22 : 0.577 & Signature.24 : 0.096

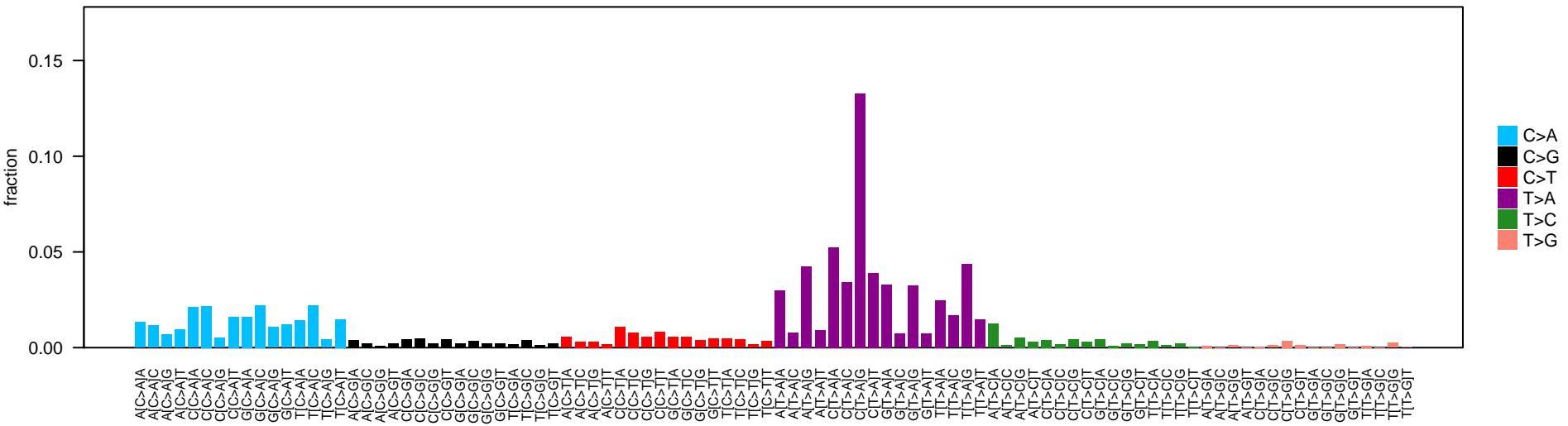

error = 0.111

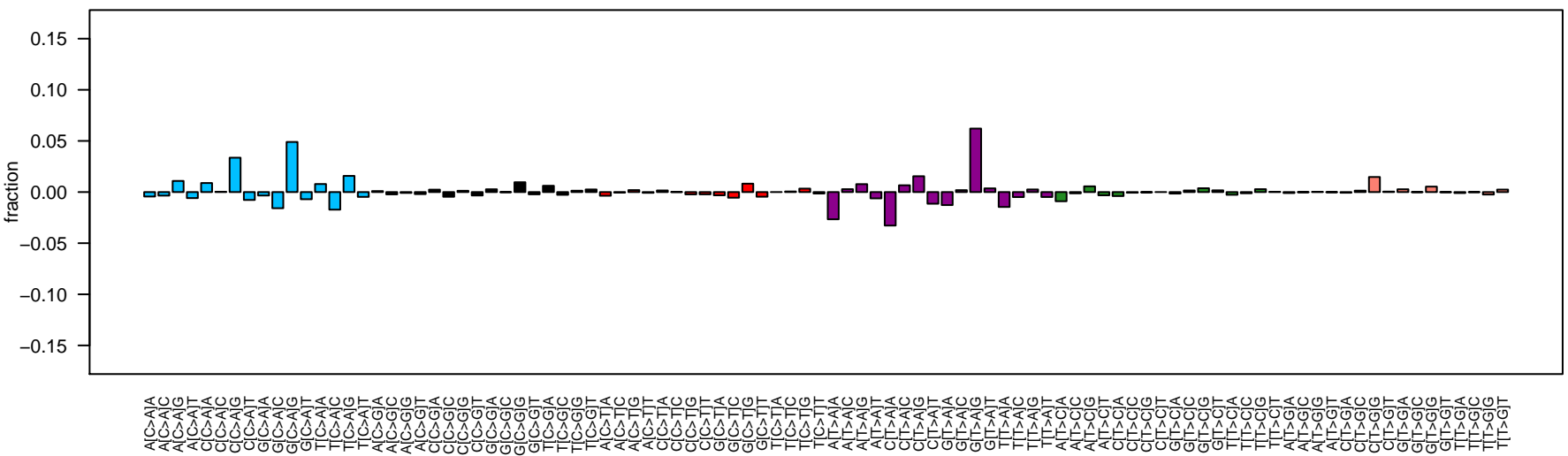

Supplement: Supplementary file 8 — Mutational signatures for all MPA/DMBA-induced tumors. (ZIP 142 kb) [file 13058_2019_1170_MOESM8_ESM.zip › S160_14_2.pdf]

**S176\_14\_2**

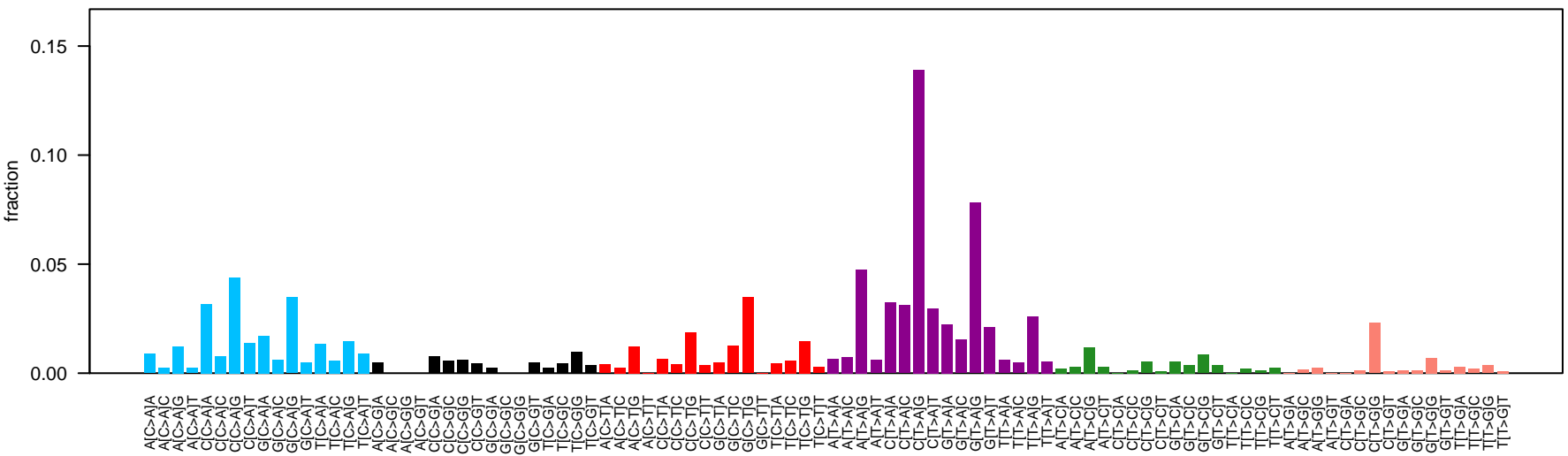

**Signature.6 : 0.117 & Signature.22 : 0.52 & Signature.24 : 0.151 & Signature.25 : 0.168**

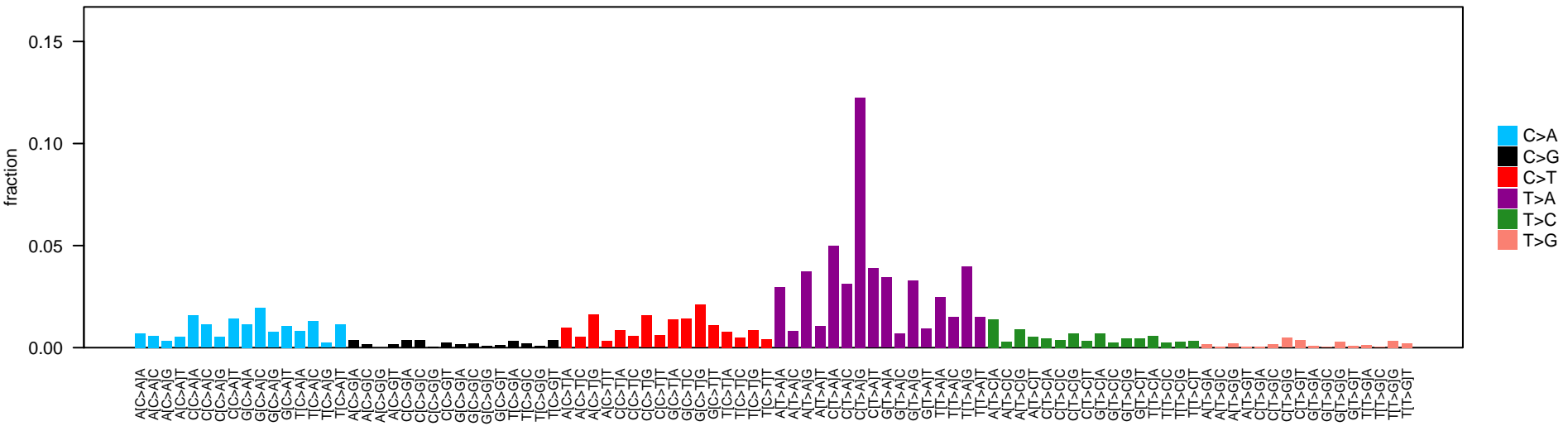

**error = 0.094**

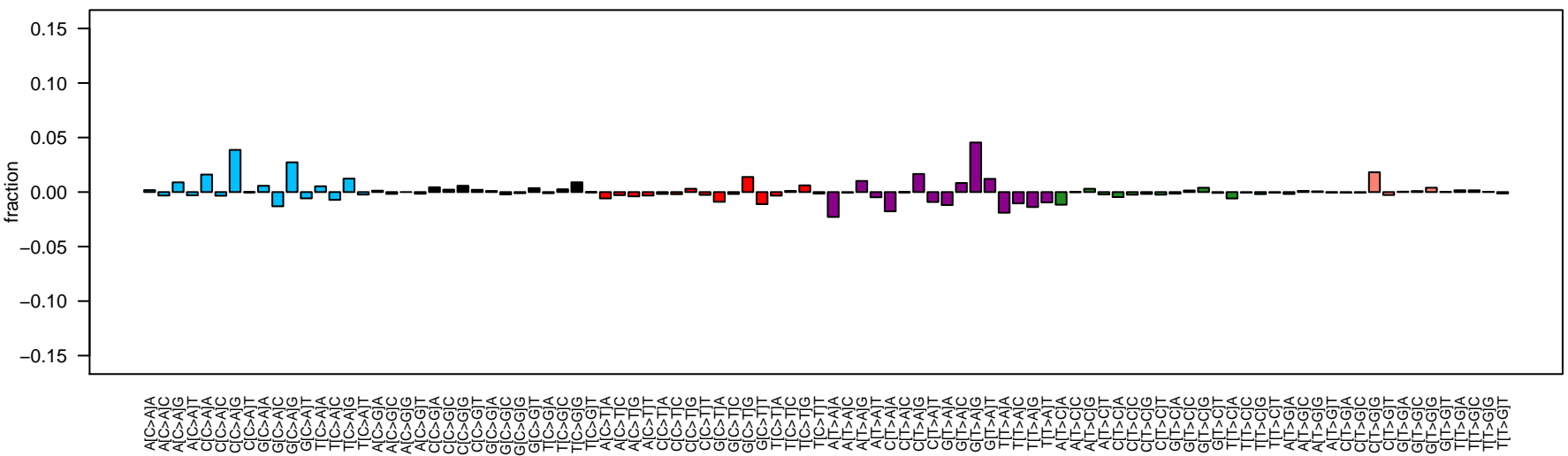

Supplement: Supplementary file 8 — Mutational signatures for all MPA/DMBA-induced tumors. (ZIP 142 kb) [file 13058_2019_1170_MOESM8_ESM.zip › S176_14_2.pdf]

S187\_14\_1

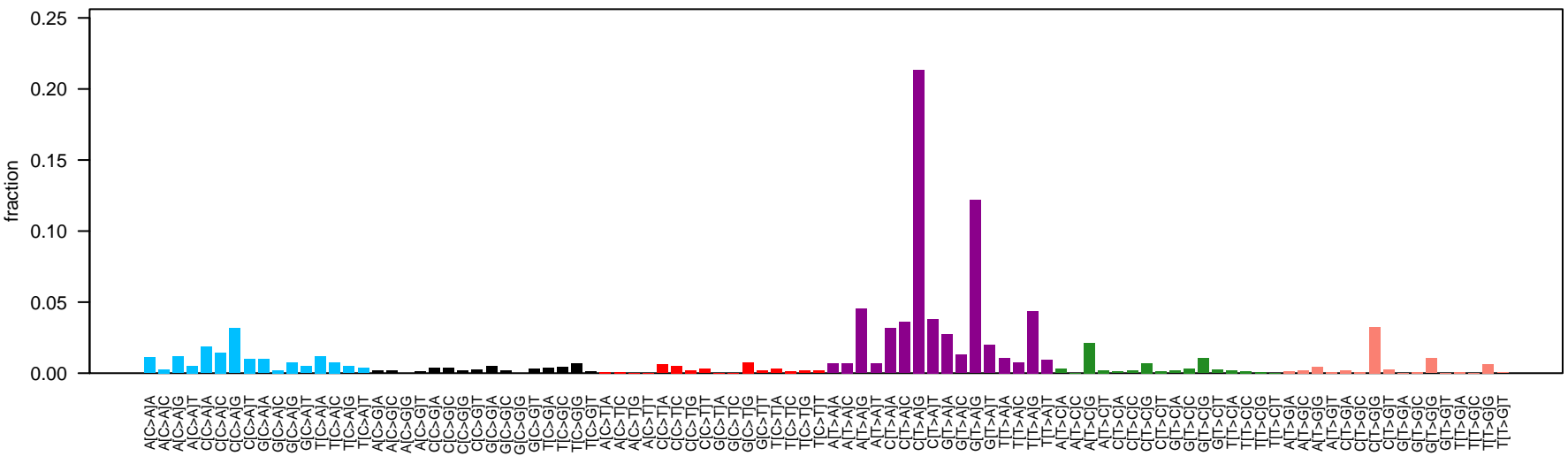

Signature.4 : 0.178 & Signature.22 : 0.792

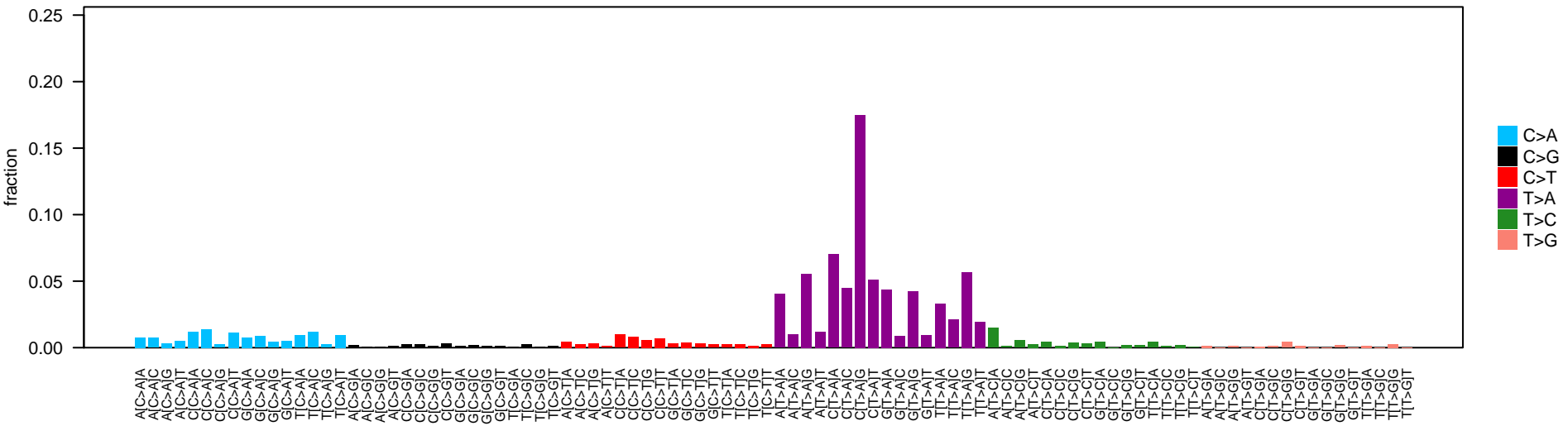

error = 0.122

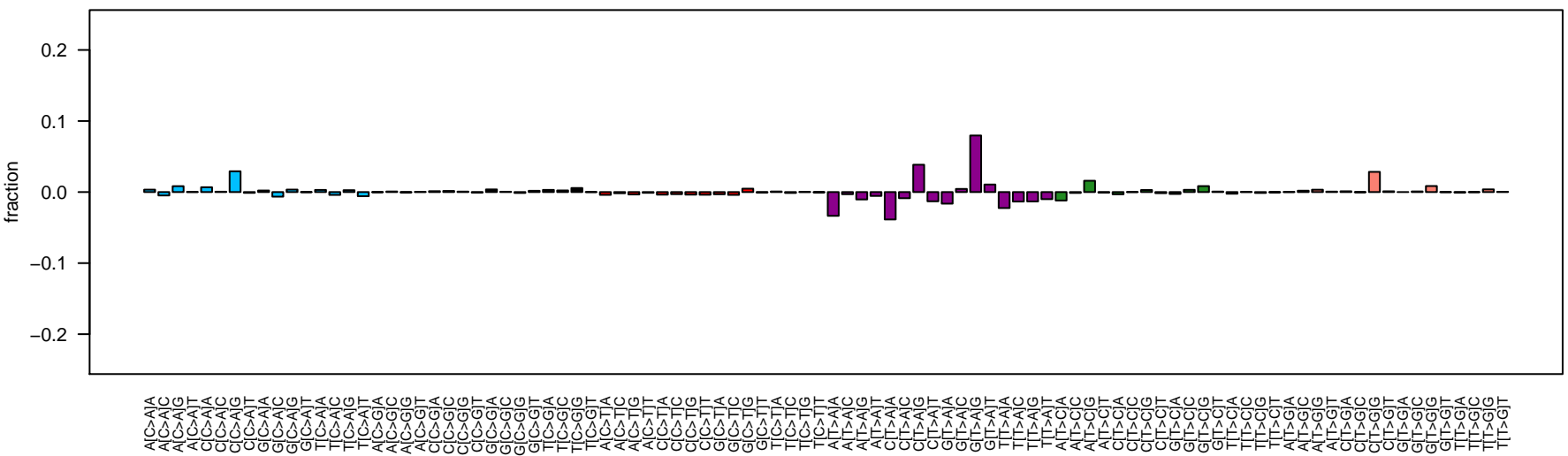

Supplement: Supplementary file 8 — Mutational signatures for all MPA/DMBA-induced tumors. (ZIP 142 kb) [file 13058_2019_1170_MOESM8_ESM.zip › S187_14_1.pdf]

S189\_14\_4

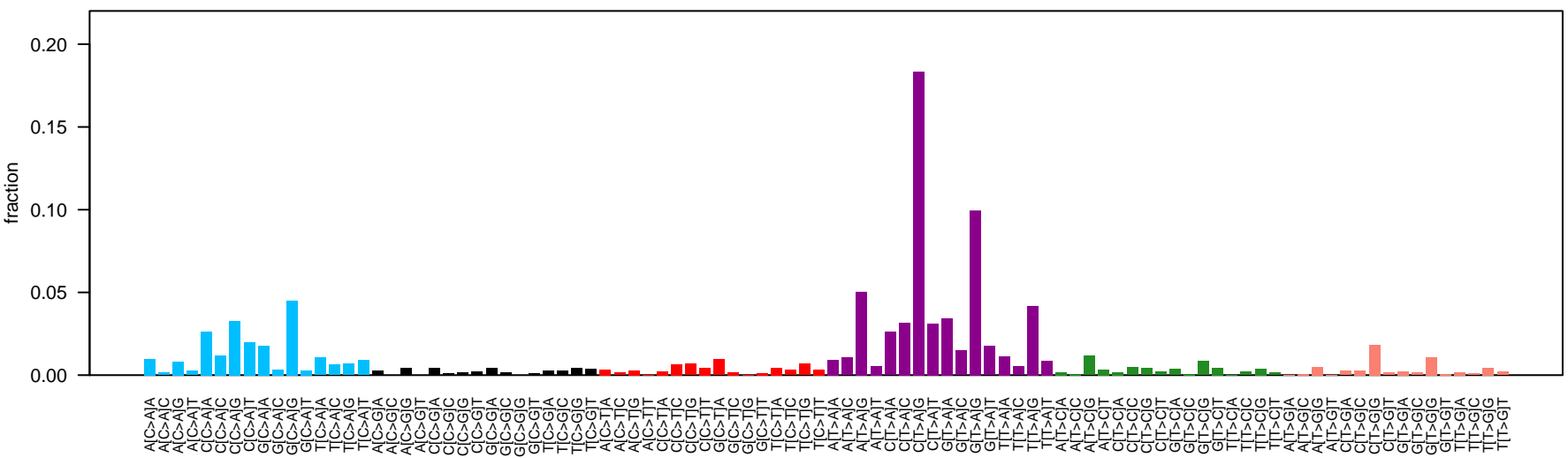

Signature.4 : 0.266 &amp; Signature.22 : 0.667 &amp; Signature.25 : 0.067

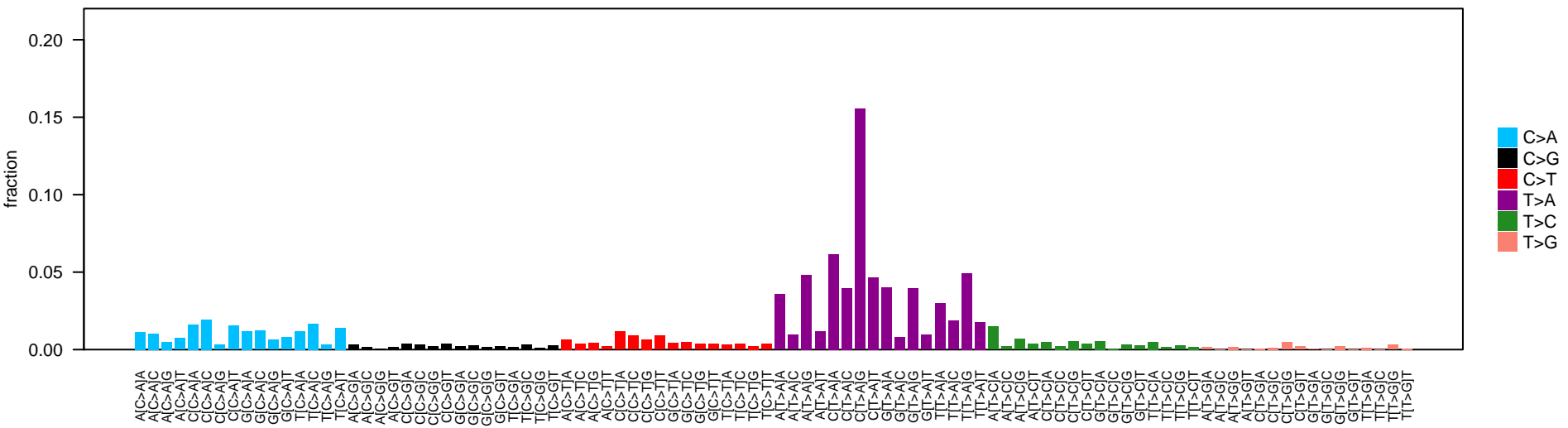

error = 0.106

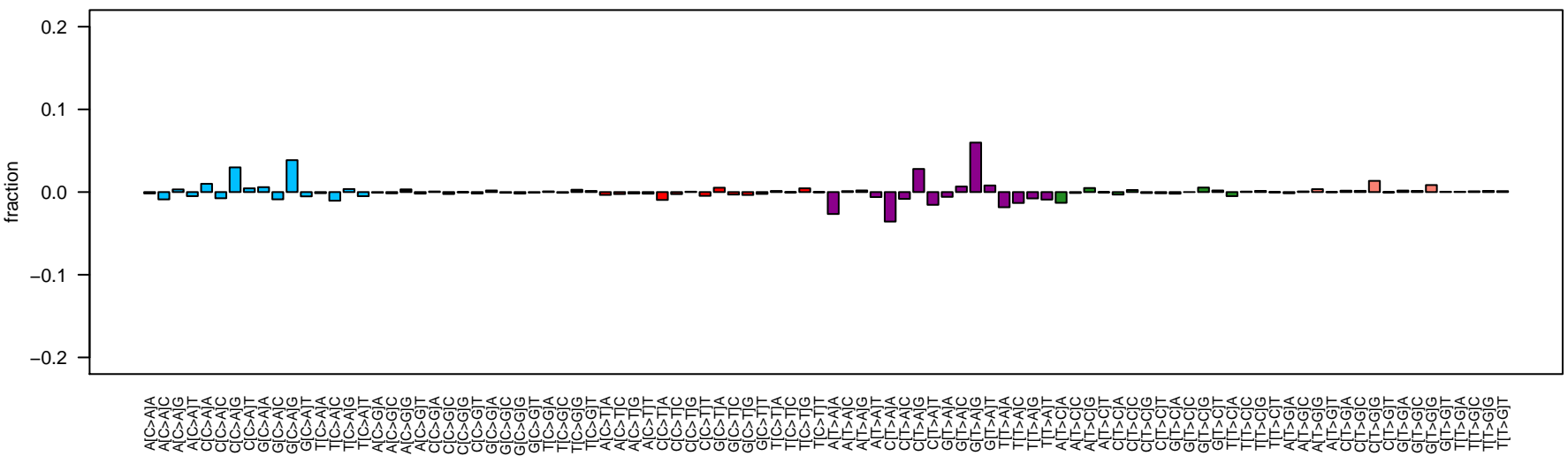

Supplement: Supplementary file 8 — Mutational signatures for all MPA/DMBA-induced tumors. (ZIP 142 kb) [file 13058_2019_1170_MOESM8_ESM.zip › S189_14_4.pdf]

S400\_15\_2

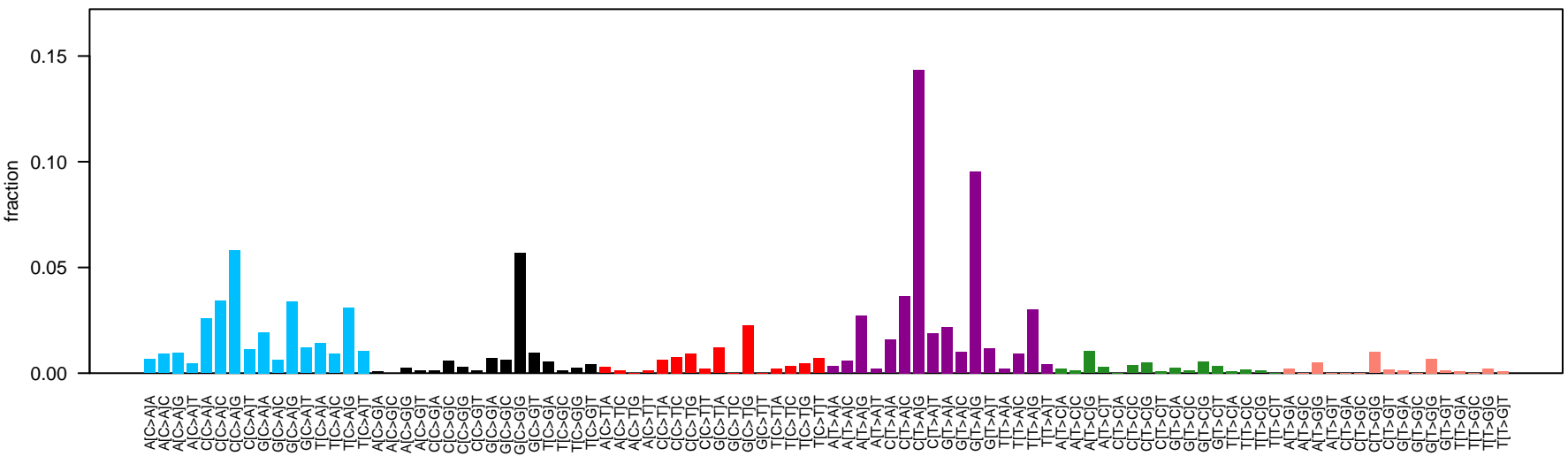

Signature.4 : 0.371 &amp; Signature.22 : 0.49 &amp; Signature.25 : 0.077

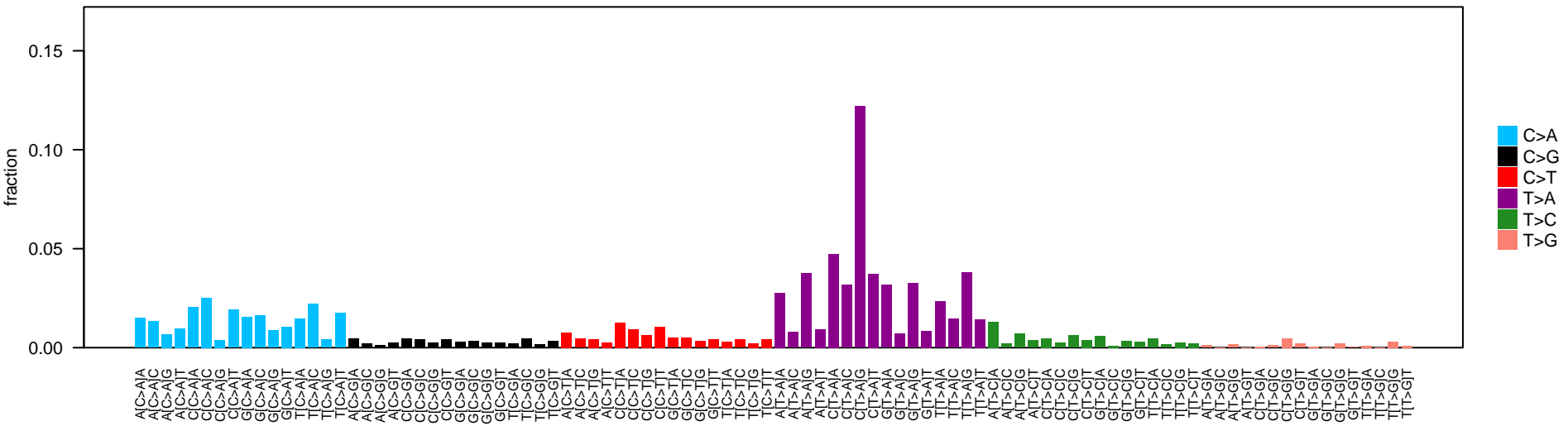

error = 0.128

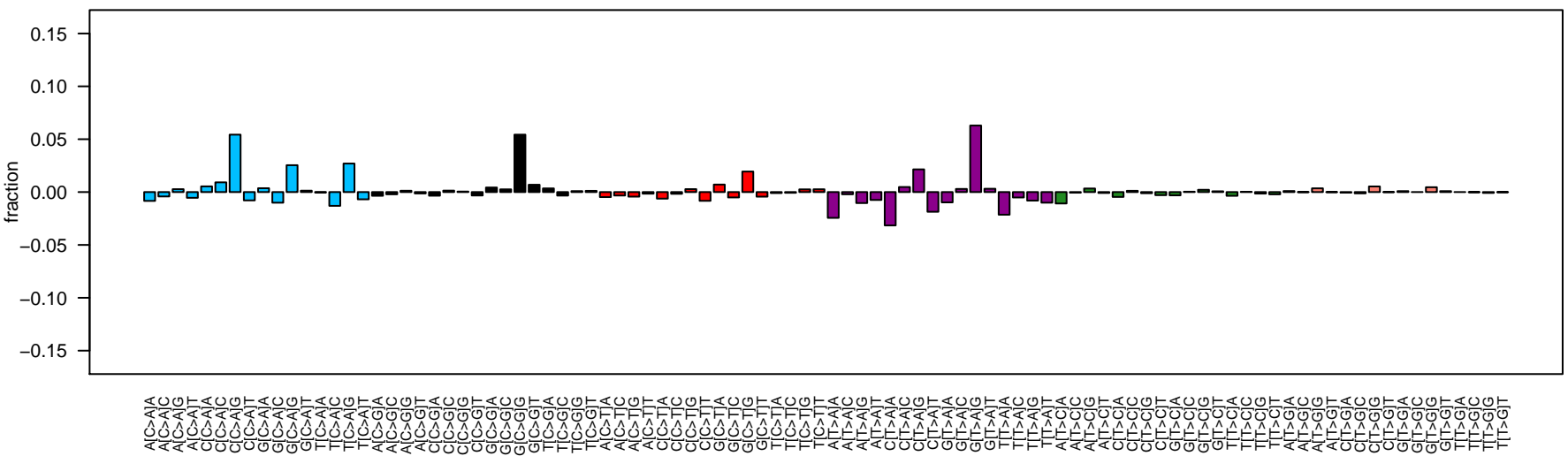

Supplement: Supplementary file 8 — Mutational signatures for all MPA/DMBA-induced tumors. (ZIP 142 kb) [file 13058_2019_1170_MOESM8_ESM.zip › S400_15_2.pdf]

**S400\_15\_7**

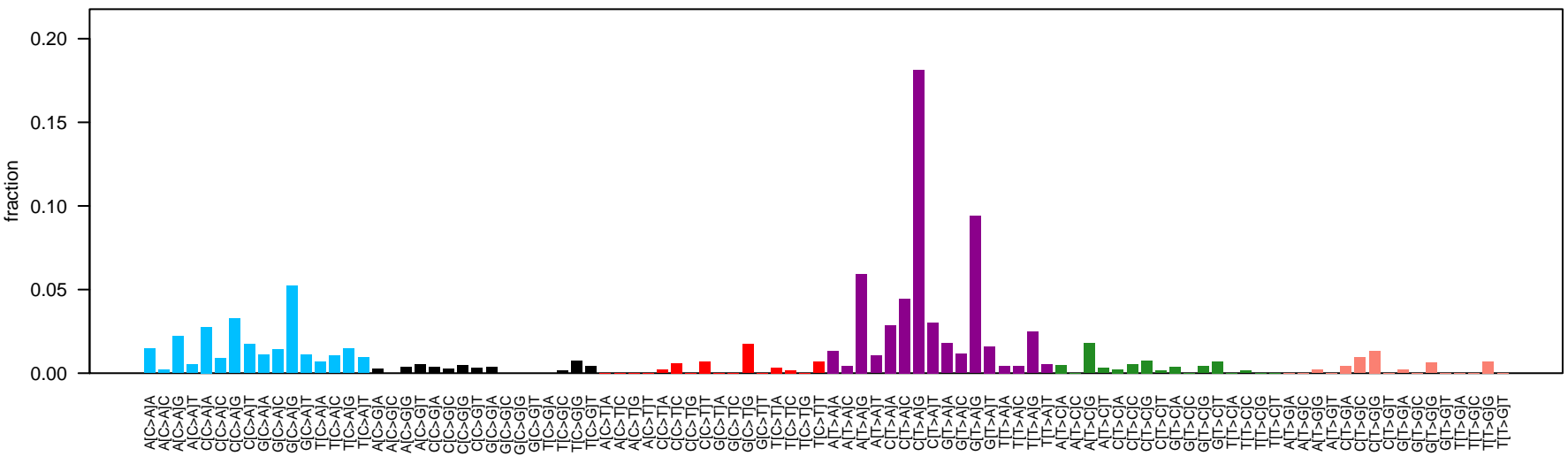

**Signature.4 : 0.283 & Signature.22 : 0.664**

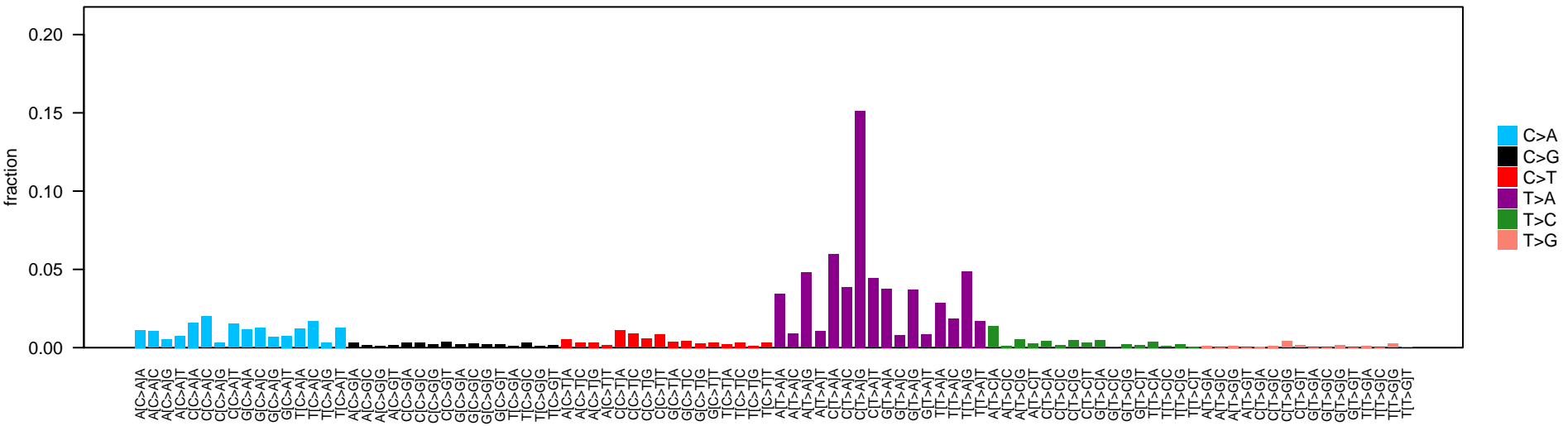

**error = 0.113**

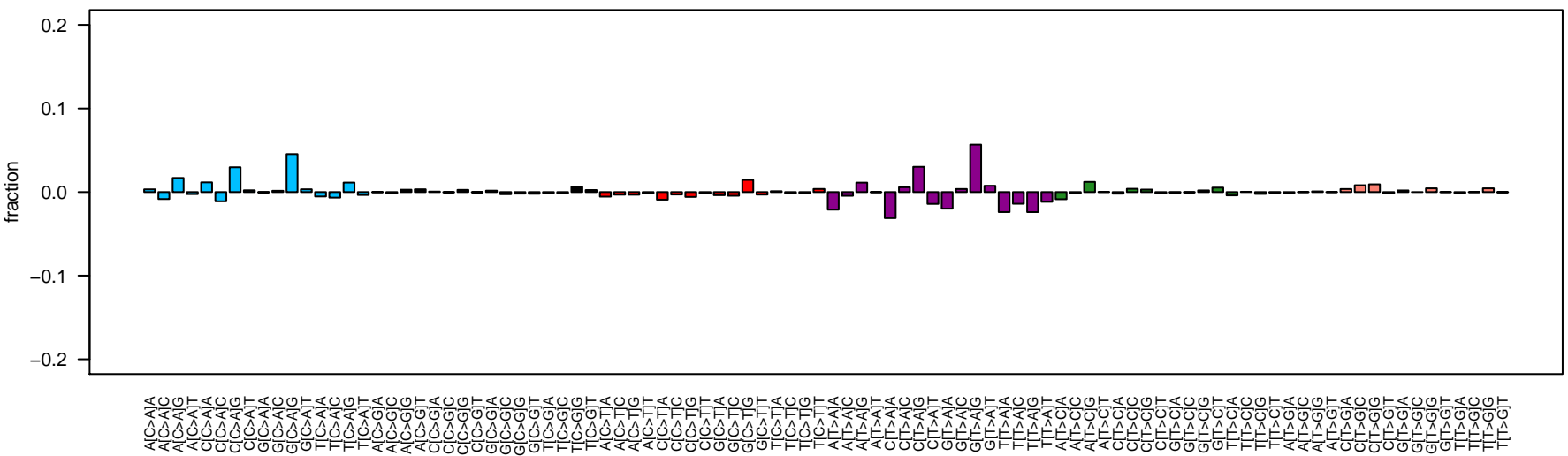

Supplement: Supplementary file 8 — Mutational signatures for all MPA/DMBA-induced tumors. (ZIP 142 kb) [file 13058_2019_1170_MOESM8_ESM.zip › S400_15_7.pdf]

S401\_15\_2

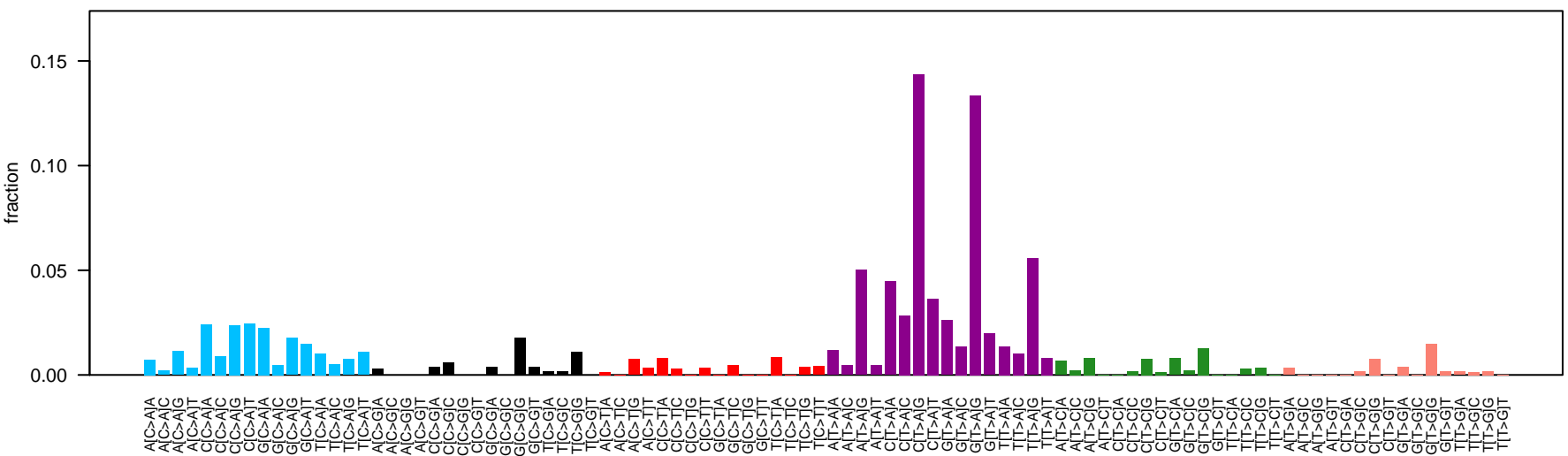

Signature.4 : 0.181 & Signature.22 : 0.594 & Signature.25 : 0.191

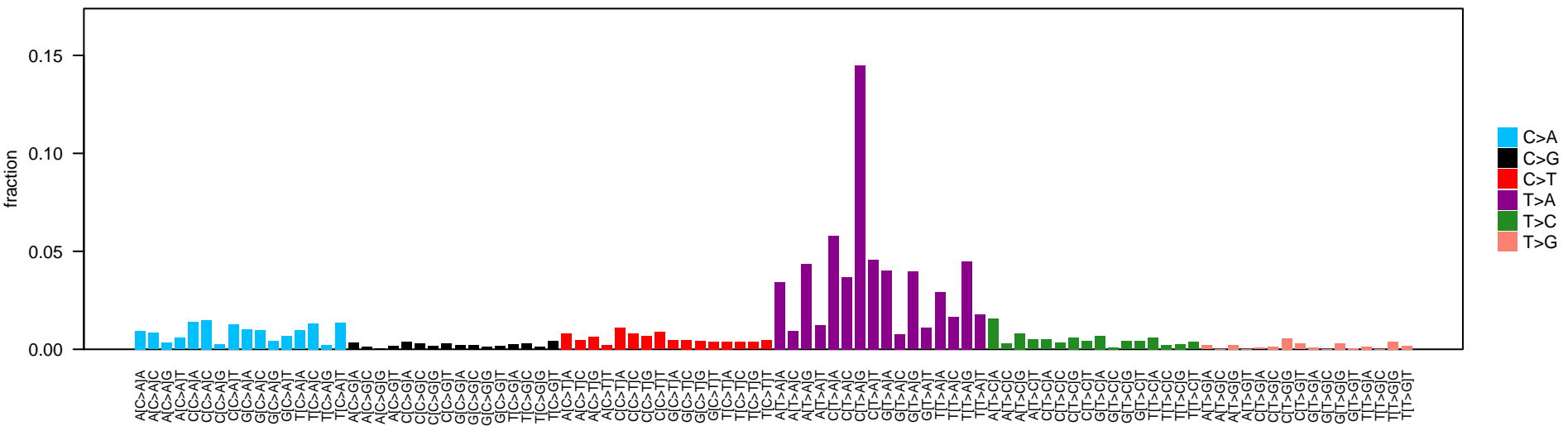

error = 0.115

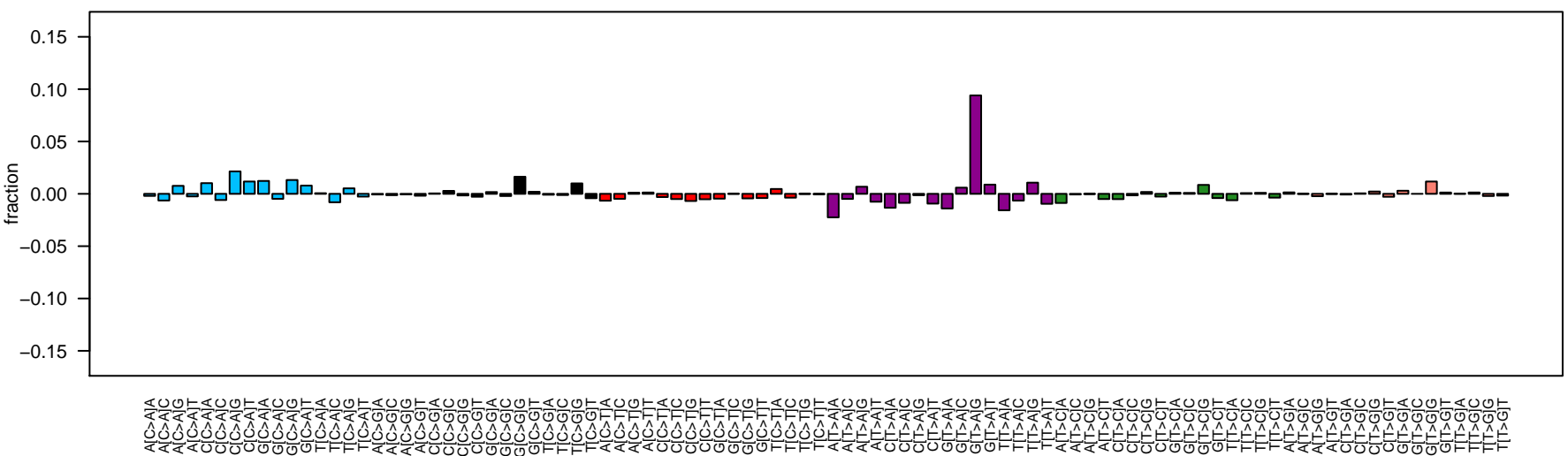

Supplement: Supplementary file 8 — Mutational signatures for all MPA/DMBA-induced tumors. (ZIP 142 kb) [file 13058_2019_1170_MOESM8_ESM.zip › S401_15_2.pdf]

**S412\_15\_2**

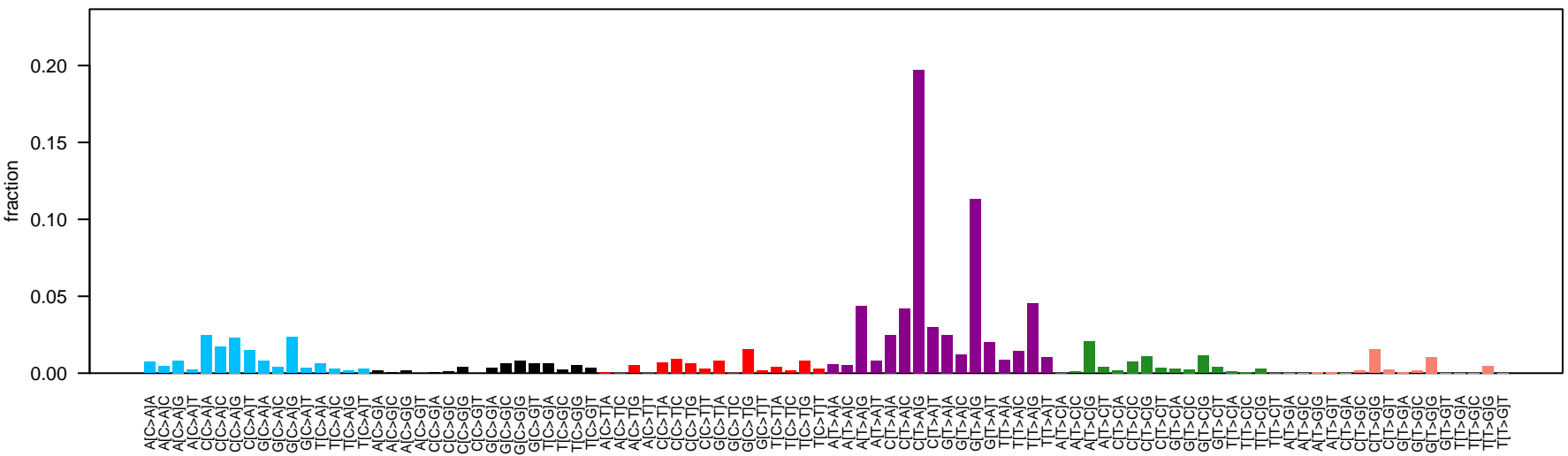

**Signature.4 : 0.2 & Signature.22 : 0.717**

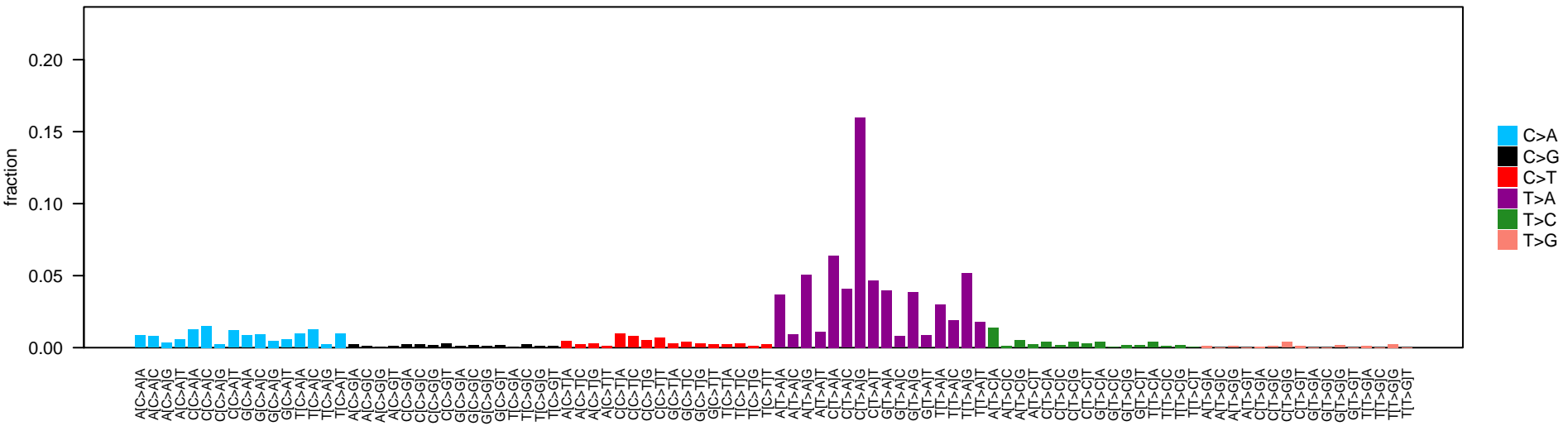

**error = 0.115**

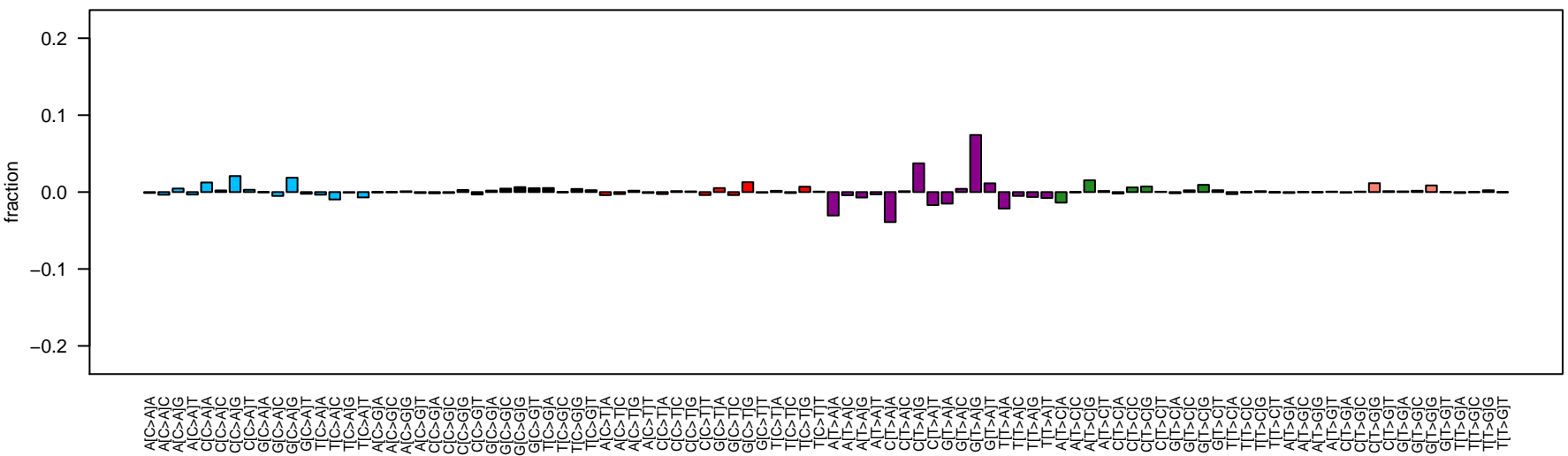

Supplement: Supplementary file 8 — Mutational signatures for all MPA/DMBA-induced tumors. (ZIP 142 kb) [file 13058_2019_1170_MOESM8_ESM.zip › S412_15_2.pdf]

**S422\_15\_2**

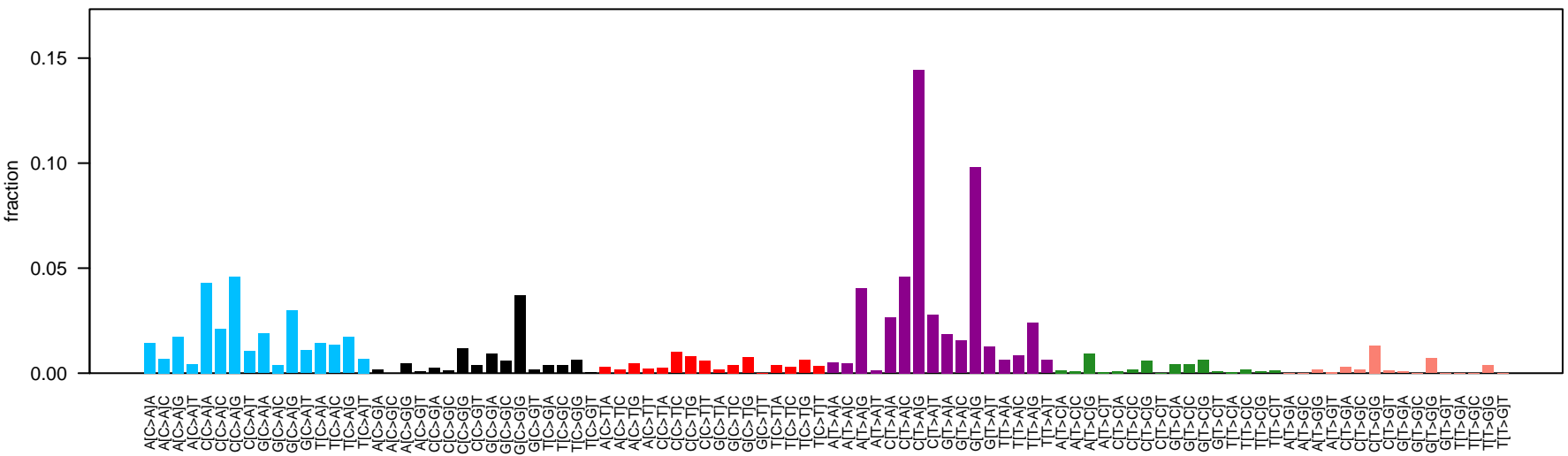

**Signature.4 : 0.382 & Signature.22 : 0.523 & Signature.25 : 0.078**

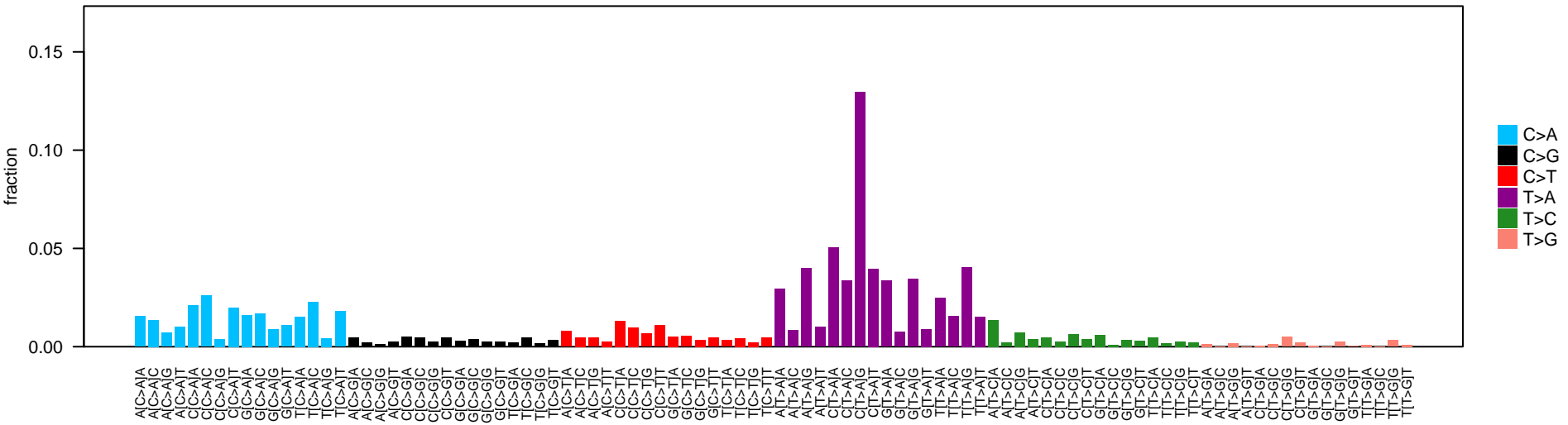

**error = 0.111**

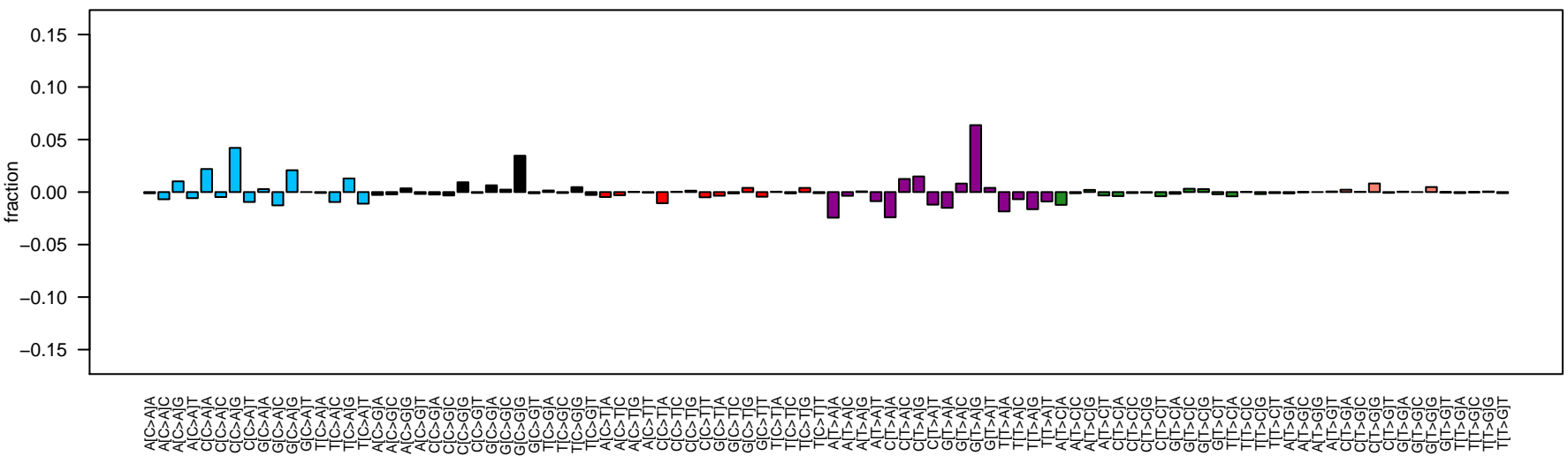

Supplement: Supplementary file 8 — Mutational signatures for all MPA/DMBA-induced tumors. (ZIP 142 kb) [file 13058_2019_1170_MOESM8_ESM.zip › S422_15_2.pdf]

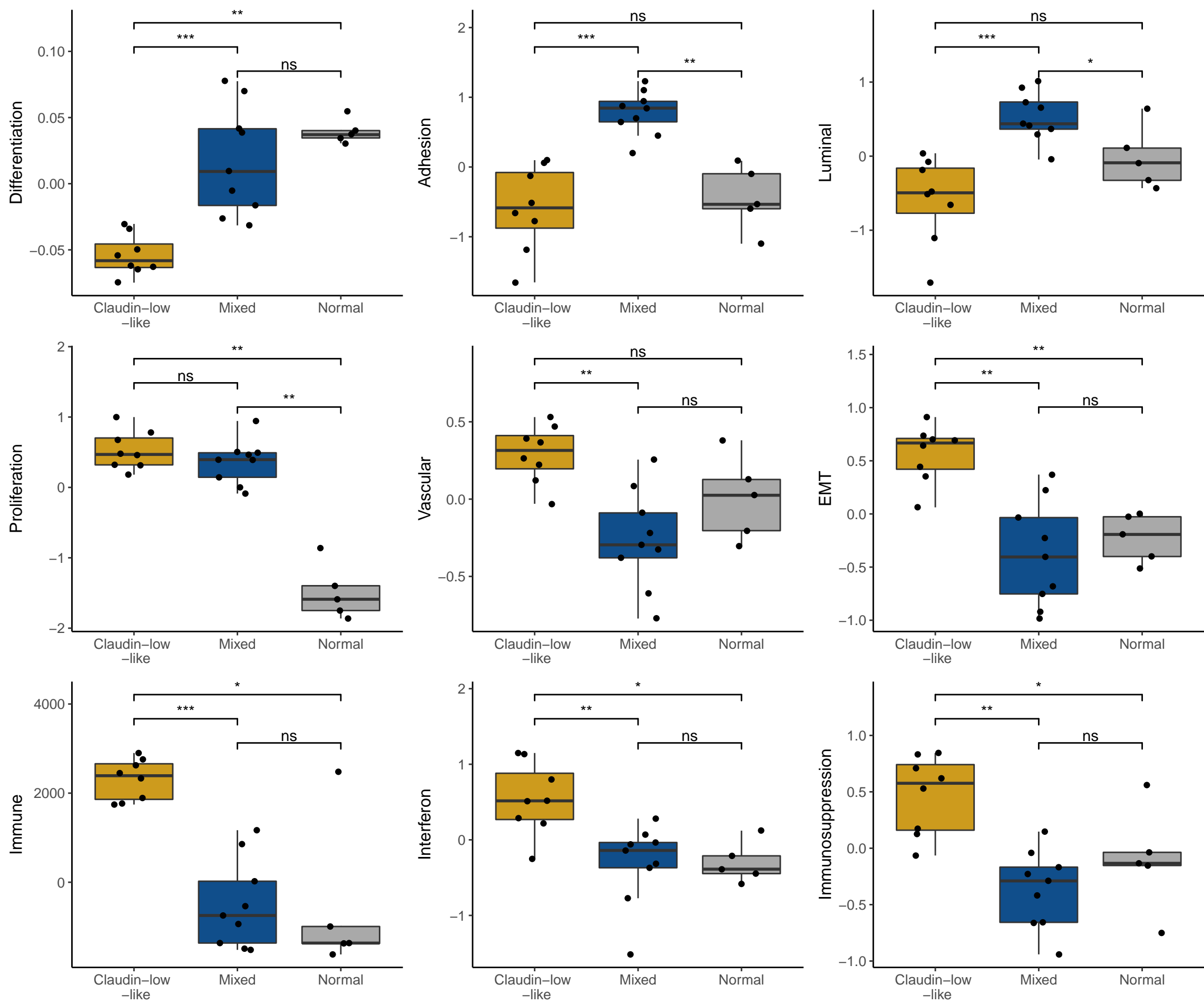

Supplement: Supplementary file 9 — Gene expression scores by cluster for genes related to differentiation, adhesion, luminal features, proliferation, vascular content, EMT, immune features, interferon signaling and immunosuppression. Two-tailed Wilcoxon rank-sum test. ns = not significant, p > 0.05. *p < 0.05. **p < 0.01. ***p < 0.001. (PDF 9 kb) [file 13058_2019_1170_MOESM9_ESM.pdf]

Cd24a

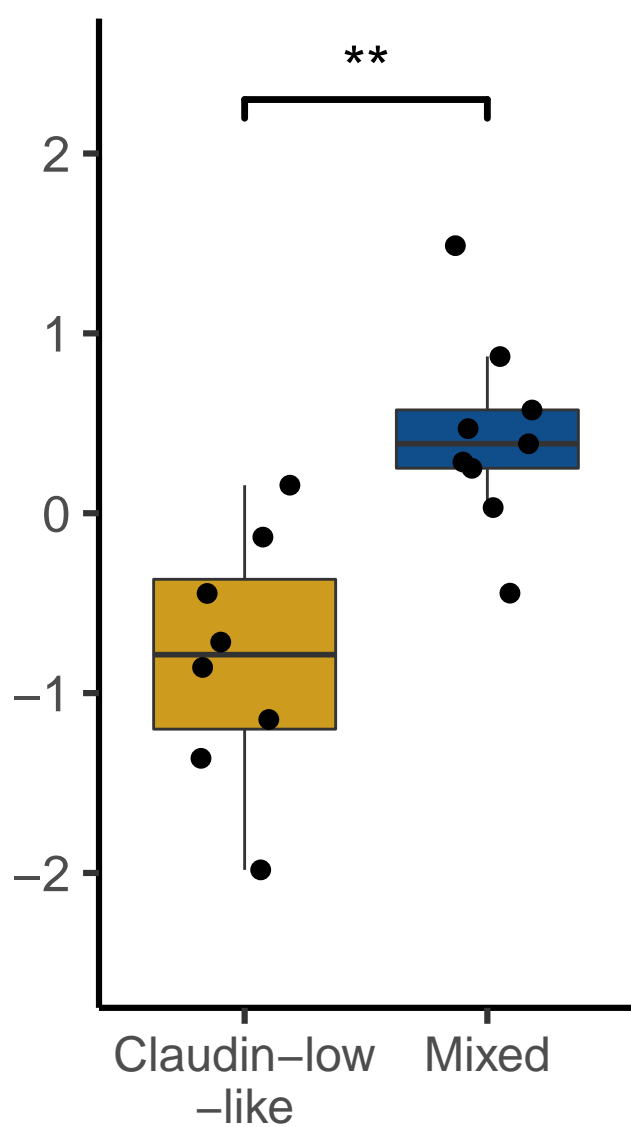

Cd44

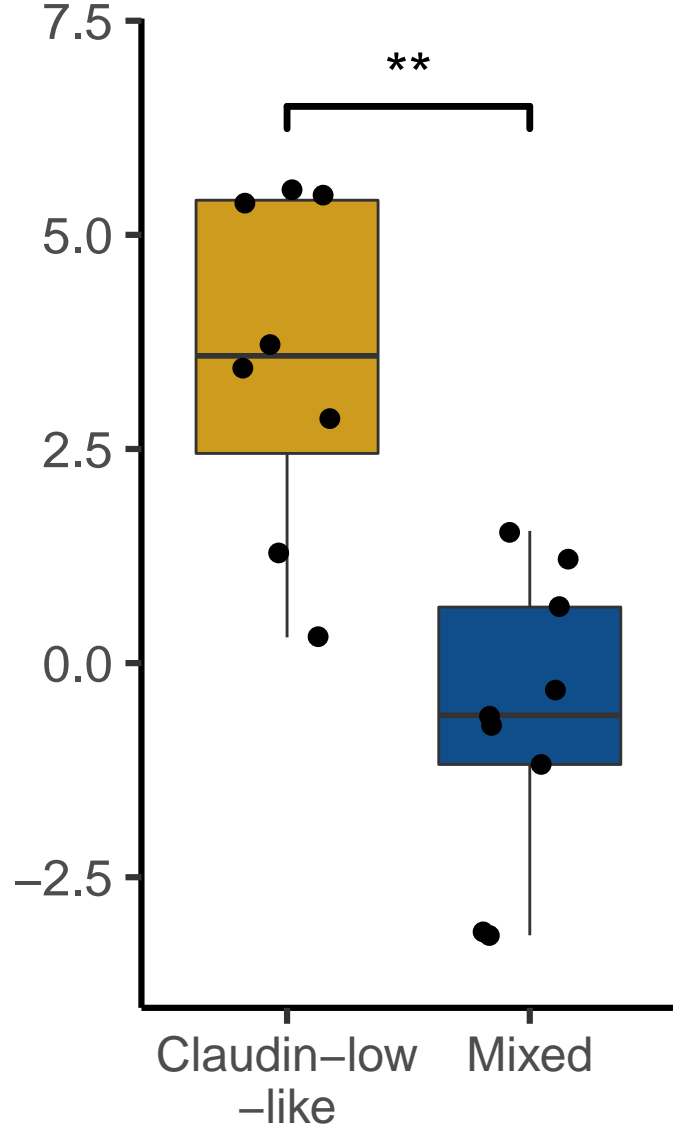

Supplement: Supplementary file 10 — Expression of Cd24a and Cd44 by cluster in MPA/DMBA-induced tumors. Claudin-low-like tumors had a lower expression of Cd24a and a higher expression of Cd44 compared to the mixed cluster of tumors (p = 0.003 and p = 0.005, respectively, two-tailed, Wilcoxon rank-sum test), indicating a stem cell-like phenotype in the claudin-low-like tumors. (PDF 5 kb) [file 13058_2019_1170_MOESM10_ESM.pdf]
