# Supplementary material for: Claudin-low-like mouse mammary tumors show distinct transcriptomic patterns uncoupled from genomic drivers
Source: Breast Cancer Res. 2019 Jul 31;21:85. doi: 10.1186/s13058-019-1170-8 (PMC6670237; doi:10.1186/s13058-019-1170-8)
Supplement: Supplementary file 8 — Mutational signatures for all MPA/DMBA-induced tumors. (ZIP 142 kb) [file S416_15_2.pdf › S416_15_2.pdf]

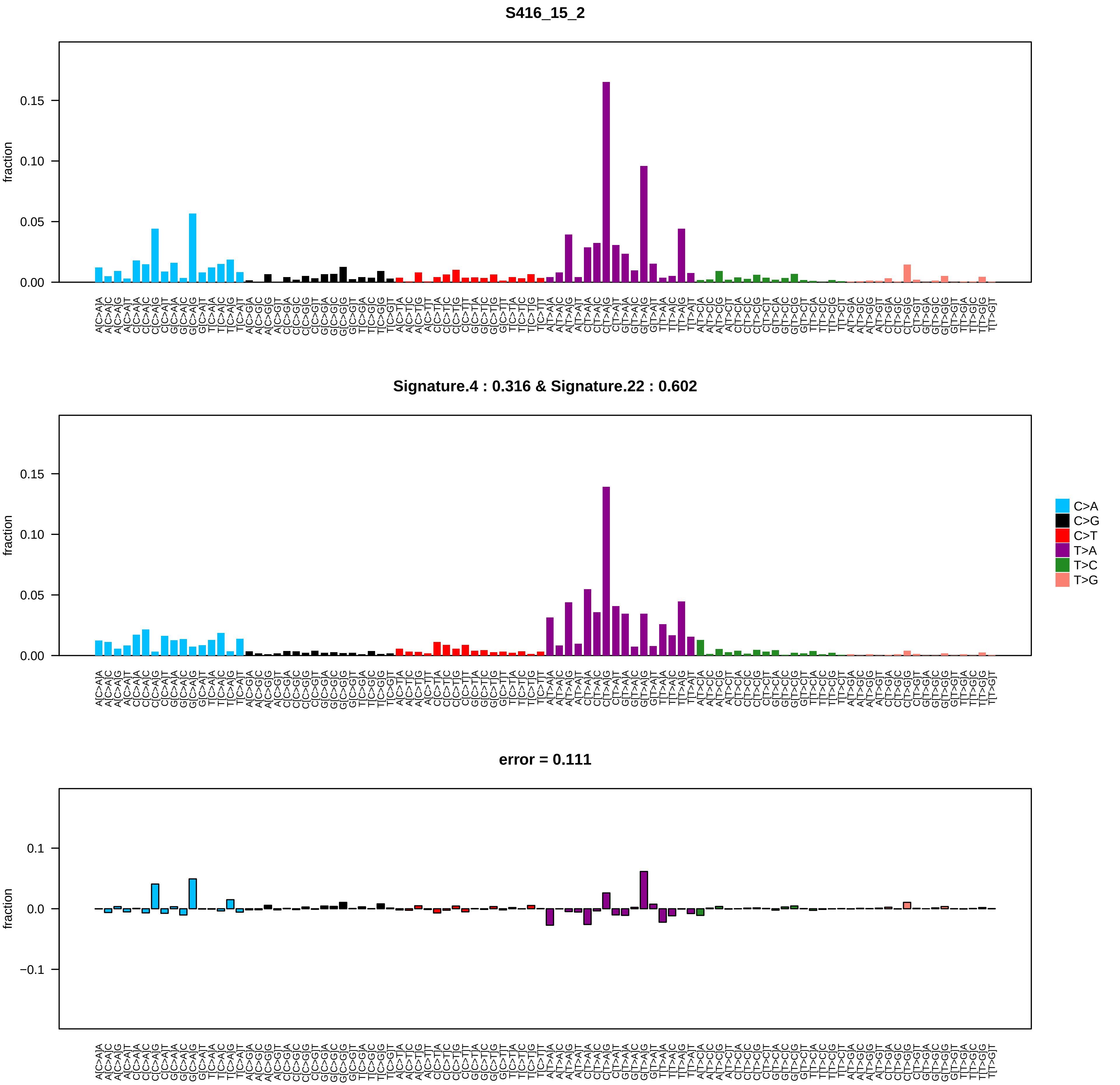

S416_15_2
fraction
0.15
0.10
0.05
0.00
Signature.4 : 0.316 & Signature.22 : 0.602
fraction
error = 0.111
0.1
0.0
−0.1
C>A
C>G
C>T
T>A
T>C
T>G
