# Supplementary material for: Claudin-low-like mouse mammary tumors show distinct transcriptomic patterns uncoupled from genomic drivers
Source: Breast Cancer Res. 2019 Jul 31;21:85. doi: 10.1186/s13058-019-1170-8 (PMC6670237; doi:10.1186/s13058-019-1170-8)
Supplement: Supplementary file 8 — Mutational signatures for all MPA/DMBA-induced tumors. (ZIP 142 kb) [file S416_15_9.pdf › S416_15_9.pdf]

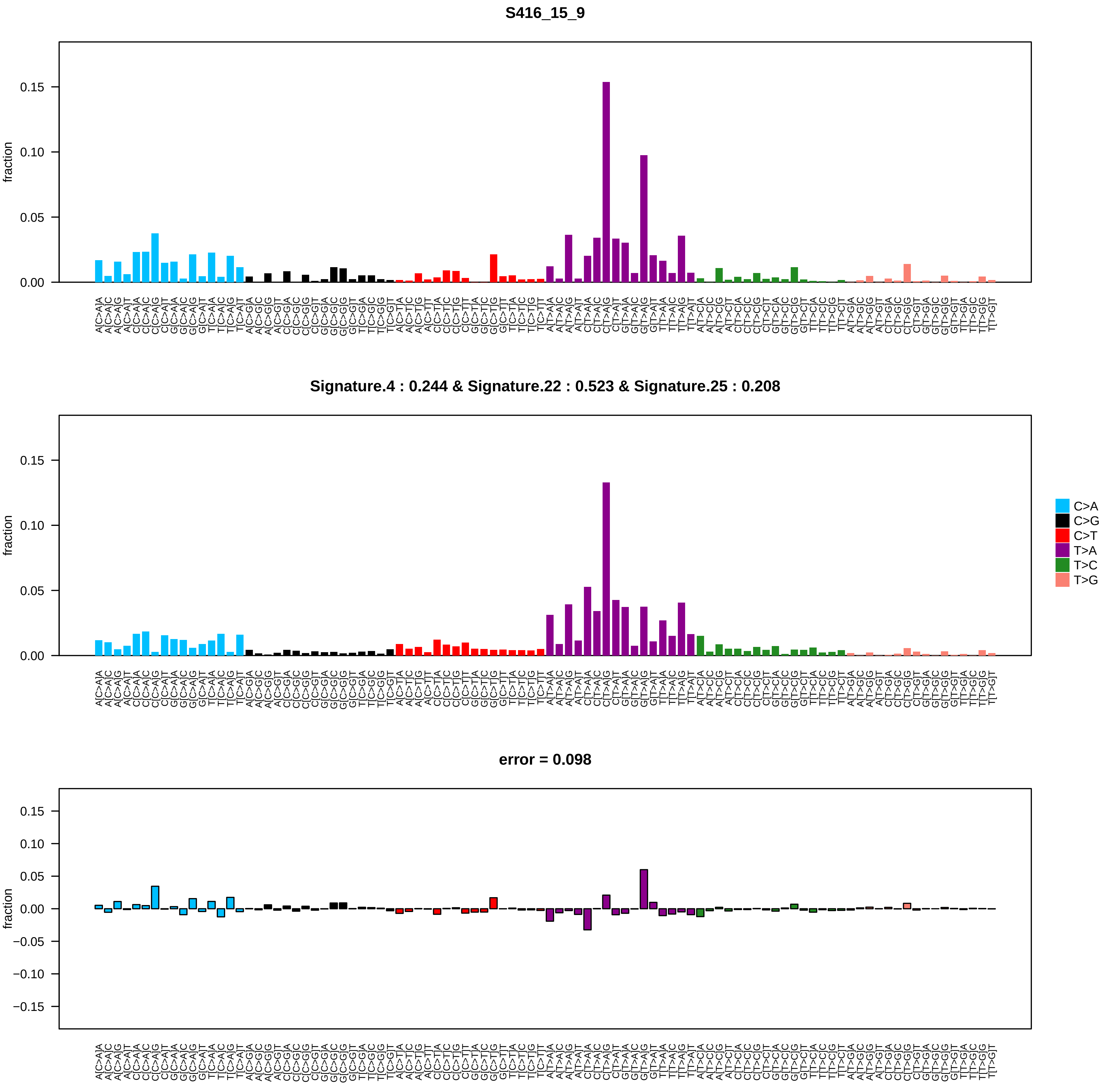

S416_15_9
Signature.4 : 0.244 & Signature.22 : 0.523 & Signature.25 : 0.208
error = 0.098
fraction
C>A
C>G
C>T
T>A
T>C
T>G
